# Supplementary material for: Novel Synthesis of Dihydroisoxazoles by p-TsOH-Participated 1,3-Dipolar Cycloaddition of Dipolarophiles withα-Nitroketones
Source: Molecules. 2023 Mar 11;28(6):2565. doi: 10.3390/molecules28062565 (PMC10052018; doi:10.3390/molecules28062565)
Supplement: Supplementary file 1 [file molecules-28-02565-s001.zip › molecules-2211321-supplementary.pdf]

Supporting Information

for

Novel synthesis of dihydroisoxazoles by *p*-TsOH-participated 1,3-dipolar cycloaddition of dipolarophiles with *α*-nitroketones

Caiyun Yang [1], Sirou Hu [1] , Xinhui Pan [1,2,*], Ke Yang[1], Ke Zhang [1], Qingguang Liu [1], Xiaobing Xin [1], Jie Li [1]，Jinhui Wang [1,] and Xiaoda Yang [1,2,*]

[1] Key Laboratory of Xinjiang Phytomedicine Resource and Utilisation, Ministry of Education, School of Pharmaceutical Sciences, Shihezi University, Shihezi 832002, China

[2] Stake Key Laboratory of Natural and Biomimetic Drugs, and Department of Chemical Biology at School of Pharmaceutical Sciences, Peking University, Beijing, 100191, China

* Correspondence: panxhshzu@shzu.edu.cn (X. P.); xyang@bjmu.edu.cn (X. Y.)

Table of Contents

1. General Experimental Methods…………………………………………....……S1

2. General procedure for the *α*-Nitroketones………………………………………S1

3. General procedure for the cycloaddition of alkenes and *α*-Nitroketones…...…S2

4. Analytical data for Products………………………………………………..…… S3

5. ^{1}H and ^{13}C NMR spectra……………………………………………………… S15

EXPERIMENTAL SECTION

General Experimental Methods. ^{1}H NMR (400 MHz) and ^{13}C NMR (101 MHz) were recorded at room temperature on DRX-400 spectrometer (Bruker, Germany) in $CDCl_3$. The chemical shifts are given in parts per million (ppm) on the delta (*δ*) scale. The solvent peak was used as a reference value, for ^{1}H NMR: $CDCl_3$ δ_H 7.26; for ^{13}C NMR: $CDCl_3$ δ_C 77.16 ppm. IR spectra were recorded using an Avatar 360 FT-IR ESP spectrometer (Nicolet, USA) at room temperature. HR-ESI-MS spectra were acquired using an Agilent 6210 ESI/TOF mass spectrometer (Agilent Technologies, Santa Clara, CA, USA). Analytical TLC was run on silica gel plates (GF254, Yantai Institute of Chemical Technology, Yantai, China). Spots on the plates were observed under UV light. Column chromatography was performed on silica gel (200~300 mesh and 300~400 mesh; Qingdao Marine Chemical Factory, Qingdao, China). Super-dry solvent *i*-PrOH, ACN, DMSO and DMF were purchased from Aldrich and used as supplied. The *α*-nitroketones were synthesized using the same method as reported in the literature[1].

General procedure for the *α*-Nitroketones.

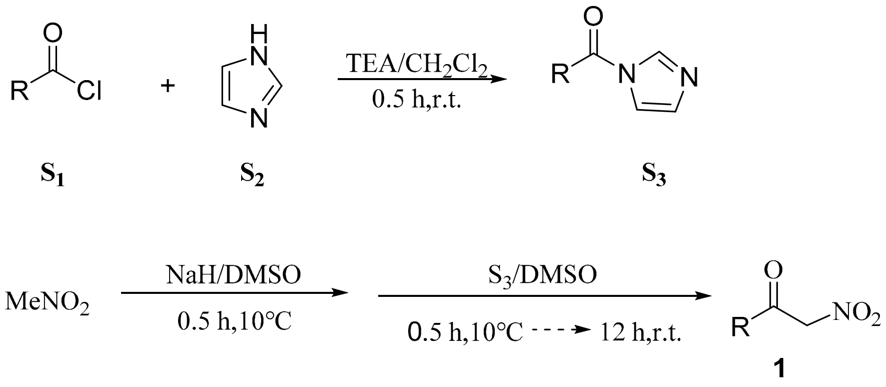

Figure S1. General procedure for the *α*-Nitroketones.

Step 1: Preparation of **S3**

Imidazole (**S2**, 18 mmol) and triethylamine (18 mmol) were dissolved in dry CH_2Cl_2 60 mL and different substituted chloride (**S1**, 15 mmol) was slowly added to the stirred solution. The mixture was stirred at room temperature for 0.5 h. Next, the mixture was quickly extracted three times with cold water and dried with anhydrous magnesium sulfate, filtered and concentrated under reduced pressure to give crude **S3**. The crude **S3** can be used for the synthesis of *α*-Nitroketones.

Step 2: Preparation of *α*-Nitroketones (**1**)

Firstly, a mixture of $MeNO_2$ (15 mmol) and NaH (33 mmol) in DMSO (60 mL) was stirred for 0.5 h while maintaining the temperature at 10 °C, and then crude

S_3 (15 mmol) was dissolved in DMSO 60 mL and slowly added at 10 °C, the mixture was stirred for 30 min, next followed by 12 h at room temperature. Subsequently, the mixture was quenched with 1 mol/L HCl (200 mL). Next, the reaction mixture was extracted with ethyl acetate and water, dried with anhydrous magnesium sulfate, filtered and concentrated under reduced pressure. Finally, the mixture was directly purified by flash chromatography (with ethyl acetate/petroleum ether as the eluent) to obtain the desired product (**1**), as shown in the Figure S1.

General procedure for the cycloaddition of alkenes and *α*-Nitroketones.

p-TsOH (0.500 mmol, 4 equiv) was added to a solution of **1** (0.125 mmol, 1 equiv) and **2** (0.625 mmol, 5 equiv) (or **4** [0.625 mmol, 5 equiv] or **6** [0.125 mmol, 1 equiv]) in ACN (0.2 mL). The mixture was then stirred at 80 ℃ until the starting material disappeared as monitored by TLC. Subsequently, the mixture was directly purified by flash chromatography (with ethyl acetate/petroleum ether as the eluent) to obtain the desired product (**3**, **5, 7** or **9**).

Analytical data for Products

2-Nitro-1-phenylethanone (1a)

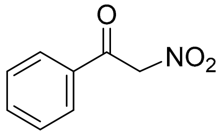

The compound was prepared following the general procedure. The title compound was isolated as a white solid, yield 76%. M.p. 99 – 101 °C, ^{1}H NMR (400 MHz, $CDCl_3$) δ_H 7.92 – 7.90 (m, 2H), 7.73 – 7.70 (tt, J = 1.3, 7.2 Hz, 1H), 7.59 – 7.55 (m, 2H), 5.92 (s, 2H). ^{1}NMR data is consistent with reported literature values[2].

1-(2-Bromophenyl)-2-nitroethanone (1b)

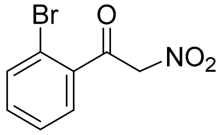

The compound was prepared following the general procedure. The title compound was isolated as a white solid, yield 77%. M.p. 47 – 48 °C, ^{1}H NMR (400 MHz, $CDCl_3$) δ_H 7.71 – 7.67 (m, 1H), 7.61 (dd, J = 7.4, 1.7 Hz, 1H), 7.45 (td, J = 6.8, 1.2 Hz, 2H), 5.88 (s, 2H). ^{1}NMR data is consistent with reported literature values[2].

1-(3-Bromophenyl)-2-nitroethanone (1c)

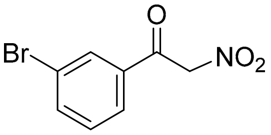

The compound was prepared following the general procedure. The title compound was isolated as a white solid, yield 77%. M.p. 100 – 102 °C, ^{1}H NMR (400 MHz, DMSO-d_6) δ_H 8.18 – 7.98 (t, J = 1.9 Hz, 1H), 8.00 – 7.87 (m, 2H), 7.65 – 7.44 (t, J = 7.9 Hz, 1H), 6.64 – 6.47 (s, 2H). ^{1}NMR data is consistent with reported literature values[2].

1-(4-Bromophenyl)-2-nitroethanone (1d)

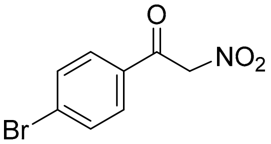

The compound was prepared following the general procedure. The title compound was isolated as a white solid, yield 71%. M.p. 185 – 188 °C, ^{1}H NMR (400 MHz, DMSO-

*d*6) δ_H 7.89 – 7.85 (m, 2H), 7.85 – 7.81 (m, 2H), 6.56 (s, 2H). [1]NMR data is consistent with reported literature values[2].

1-(4-Methylphenyl)-2-nitroethanone (1e)

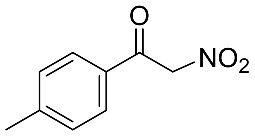

The compound was prepared following the general procedure. The title compound was isolated as a white solid, yield 88%. M.p. 143 – 145 °C, ^{1}H NMR (400 MHz, DMSO-*d*6) δ_H 7.86 – 7.82 (m, 2H), 7.43 – 7.38 (m, 2H), 6.53 (s, 2H), 2.41 – 2.40 (s, 3H). [1]NMR data is consistent with reported literature values[2].

1-(4-Methoxyphenyl)-2-nitroethanone (1f)

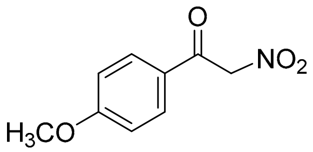

The compound was prepared following the general procedure. The title compound was isolated as a white solid, yield 73%. M.p. 157 – 160 °C, ^{1}H NMR (400 MHz, DMSO-*d*6) δ_H 7.94 – 7.90 (m, 2H), 7.14 – 7.09 (m, 2H), 6.40 (s, 2H), 3.87 (s, 1H). [1]NMR data is consistent with reported literature values[2].

1-[4-(1,1-Dimethylethyl)phenyl]-2-nitroethanone (1g)

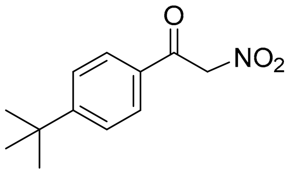

The compound was prepared following the general procedure. The title compound was isolated as a white solid, yield 99%. M.p. 50 – 51 °C, ^{1}H NMR (400 MHz, DMSO-*d*6) δ_H 7.91 – 7.90 (m, 1H), 7.89 – 7.88 (m, 1H), 7.63 – 7.61 (m, 1H), 7.61 – 7.59 (m, 1H), 6.51 (s, 2H), 1.31 (s, 9H). [1]NMR data is consistent with reported literature values[2].

1-[1,1′-Biphenyl]-4-yl-2-nitroethanone (1h)

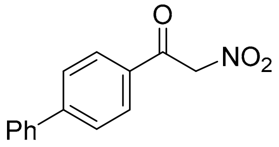

The compound was prepared following the general procedure. The title compound was

isolated as a white solid, yield 78%. M.p. 130 – 132 °C, ^{1}H NMR (400 MHz, DMSO-d_6) δ_H 8.05 – 8.01 (m, 2H), 7.93 – 7.89 (m, 2H), 7.80 – 7.77 (m, 2H), 7.55 – 7.50 (m, 2H), 7.48 – 7.43 (m, 1H), 6.58 (s, 2H). ^{1}NMR data is consistent with reported literature values[2].

1-(4-Fluorophenyl)-2-nitroethanone (1i)

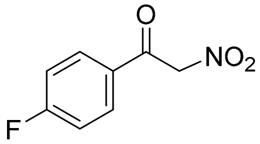

The compound was prepared following the general procedure. The title compound was isolated as a white solid, yield 85%. M.p. 67-71 °C, ^{1}H NMR (400 MHz, $CDCl_3$) δ_H 7.99 – 7.94 (m, 2H), 7.32 – 7.28 (m, 1H), 7.25 – 7.22 (m, 1H), 5.90 (s, 2H). ^{1}NMR data is consistent with reported literature values[3].

(5-Benzyl-4,5-dihydroisoxazol-3-yl)(phenyl)methanone (3a)

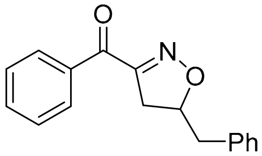

The compound was prepared following the general procedure. The title compound was isolated as a pale yellow oily liquid, yield 90%. ^{1}H NMR (400 MHz, $CDCl_3$) δ_H 8.19 – 8.12 (m, 2H), 7.63 – 7.57 (m, 1H), 7.50 – 7.44 (m, 2H), 7.38 – 7.27 (m, 5H), 5.12 – 5.03 (m, 1H), 3.37 (dd, J =17.6, 10.8 Hz, 1H), 3.17 – 3.09 (m, 2H), 2.98 (dd, J = 14.0, 6.5 Hz, 1H). ^{13}C NMR (101 MHz, $CDCl_3$) δ_C 186.2, 157.5, 135.8, 135.6, 133.3, 130.1 (2C), 129.3 (2C), 128.5 (2C), 128.1 (2C), 126.8, 83.3, 40.7, 38.1; IR νmax 3033, 1654, 1581, 710, 672 cm^{-1}; HRMS (EI) *m/z* calcd for $C_{17}H_{16}NO_2$ $[M + H]^+$ 266.1176, found 266.1178. These data are consistent with reported literature values[4,5].

(5-Benzyl-4,5-dihydroisoxazol-3-yl)(2-bromophenyl)methanone (3b)

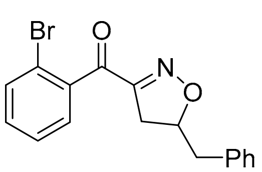

The compound was prepared following the general procedure. The title compound was isolated as a pale yellow oily liquid, yield 73%. ^{1}H NMR (400 MHz, $CDCl_3$) δ_H 7.51 (d, J = 7.6 Hz, 1H), 7.28 – 7.25 (m, 2H), 7.25 –7.23 (m, 2H), 7.22 –7.20 (m, 1H),

7.20–7.14 (m, 3H), 5.05 (ddt, J = 11.0, 7.6, 6.3 Hz, 1H), 3.22 (dd, J = 17.5, 10.9 Hz, 1H), 3.06 – 2.94 (m, 2H), 2.88 (dd, J = 14.0, 6.5 Hz, 1H); ^{13}C NMR (101 MHz, $CDCl_3$) δ_C 189.2, 158.1, 139.2, 135.8, 133.4, 132.1, 129.8, 129.7 (2C), 128.8 (2C), 127.2, 127.1, 120.0, 85.2, 41.0, 36.7; IR νmax 3034, 1680, 1585, 755, 694 cm^{-1}; HRMS (EI) *m/z* calcd for $C_{17}H_{15}NO_2Br$ [M + H]$^+$ 344.0281, found 344.0279. These data are consistent with reported literature values[5].

(5-Benzyl-4,5-dihydroisoxazol-3-yl)(3-bromophenyl)methanone (3c)

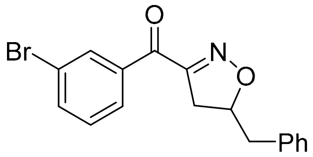

The compound was prepared following the general procedure. The title compound was isolated as a pale yellow oily liquid, yield 70%. ^{1}H NMR (400 MHz, $CDCl_3$) δ_H 8.23 (t, J = 1.9 Hz, 1H), 8.06 (dt, J = 7.8, 1.4 Hz, 1H), 7.72 – 7.68 (m, 1H), 7.36 – 7.30 (m, 3H), 7.28 – 7.24 (m, 3H), 5.13 – 5.02 (m, 1H), 3.34 (dd, J = 17.6, 10.9 Hz, 1H), 3.13 – 3.04 (m, 2H), 2.98 (dd, J = 14.0, 6.4 Hz, 1H); ^{13}C NMR (101 MHz, $CDCl_3$) δ_C 185.0, 157.6, 137.5, 136.4, 135.9, 133.2, 129.9, 129.6(2C), 128.9, 128.8(2C), 127.2, 122.6, 83.9, 40.9, 38.1; IR νmax 3038, 1691, 1616, 910, 811, 742 cm^{-1}; HRMS (EI) *m/z* calcd for $C_{17}H_{15}NO_2Br$ [M + H]$^+$ 344.0281, found 344.0293. These data are consistent with reported literature values[5].

(5-Benzyl-4,5-dihydroisoxazol-3-yl)(4-bromophenyl)methanone (3d)

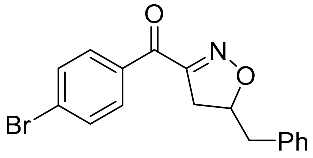

The compound was prepared following the general procedure. The title compound was isolated as a pale yellow oily liquid, yield 67%. ^{1}H NMR (400 MHz, $CDCl_3$) δ_H 8.04 – 7.97 (m, 2H), 7.63 – 7.55 (m, 2H), 7.35 – 7.30 (m, 2H), 7.28 – 7.24 (m, 3H), 5.07 (ddt, J = 10.9, 7.9, 6.3 Hz, 1H), 3.34 (dd, J = 17.6, 10.9 Hz, 1H), 3.15 – 3.04 (m, 2H), 2.96 (dd, J = 14.0, 6.5 Hz, 1H); ^{13}C NMR (101 MHz, $CDCl_3$) δ_C 185.4, 157.9, 136.1, 134.6, 132.0 (2C), 131.9 (2C), 129.7 (2C), 129.2, 128.9 (2C), 127.2, 83.9, 41.1, 38.3; IR νmax 3046, 1696, 1615, 801, 750, 686 cm^{-1}; HRMS (EI) *m/z* calcd for

$C_{17}H_{15}NO_2Br$ [M + H]$^+$ 344.0281, found 344.0276. These data are consistent with reported literature values[5].

(5-Benzyl-4,5-dihydroisoxazol-3-yl)(p-tolyl)methanone (3e)

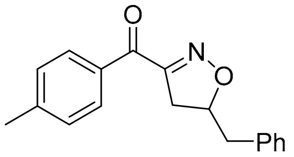

The compound was prepared following the general procedure. The title compound was isolated as a pale yellow oily liquid, yield 89%. ^{1}H NMR (400 MHz, $CDCl_3$) δ_H 8.09 – 8.01 (m, 2H), 7.37 – 7.30 (m, 2H), 7.29 – 7.22 (m, 5H), 5.05 (ddt, J = 10.8, 7.9, 6.3 Hz, 1H), 3.35 (dd, J = 17.6, 10.8 Hz, 1H), 3.16 – 3.06 (m, 2H), 2.96 (dd, J = 14.0, 6.6 Hz, 1H), 2.42 (s, 3H); ^{13}C NMR (101 MHz, $CDCl_3$) δ_C 186.1, 157.9, 144.7, 136.2, 133.4, 130.6 (2C), 129.6 (2C), 129.2 (2C), 128.8 (2C), 127.1, 83.5, 41.1, 38.6, 21.9; IR νmax 3023, 2923, 1650, 1600, 831, 750, 693 cm^{-1}; HRMS (EI) *m/z* calcd for $C_{18}H_{18}NO_2$ [M + H]$^+$ 280.1332, found 280.1336. These data are consistent with reported literature values[5].

(5-Benzyl-4,5-dihydroisoxazol-3-yl)(4-methoxyphenyl)methanone (3f)

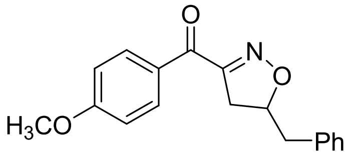

The compound was prepared following the general procedure. The title compound was isolated as a pale yellow oily liquid, yield 66%. ^{1}H NMR (400 MHz, $CDCl_3$) δ_H 8.23 – 8.15 (m, 2H), 7.36 – 7.30 (m, 2H), 7.29 – 7.25 (m, 3H), 6.97 – 6.91 (m, 2H), 5.04 (ddt, J = 10.8, 7.9, 6.4 Hz, 1H), 3.88 (s, 3H), 3.36 (dd, J = 17.6, 10.8 Hz, 1H), 3.16 – 3.07 (m, 2H), 2.96 (dd, J = 14.0, 6.6 Hz, 1H); ^{13}C NMR (101 MHz, $CDCl_3$) δ_C 184.7, 164.2, 157.9, 136.3, 132.9 (2C), 129.6 (2C), 128.8 (2C), 127.1, 113.8 (2C), 83.3, 55.7, 41.1, 38.8; IR νmax 3022, 2801, 1618, 1531, 802, 719, 676 cm^{-1}; HRMS (EI) *m/z* calcd for $C_{18}H_{18}NO_3$ [M + H]$^+$ 296.1281, found 296.1285. These data are consistent with reported literature values[5].

(5-Benzyl-4,5-dihydroisoxazol-3-yl)(4-tert-butylphenyl)methanone (3g)

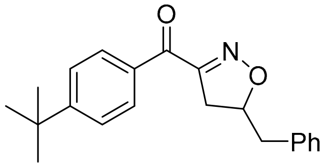

The compound was prepared following the general procedure. The title compound was isolated as a pale yellow oily liquid, yield 80%. ^{1}H NMR (400 MHz, $CDCl_3$) δ_H 8.14 – 8.09 (m, 2H), 7.53 – 7.47 (m, 2H), 7.39 – 7.24 (m, 5H), 5.06 (ddt, J = 10.8, 7.9, 6.3 Hz, 1H), 3.36 (dd, J = 17.6, 10.8 Hz, 1H), 3.19 – 3.08 (m, 2H), 2.97 (dd, J = 14.0, 6.5 Hz, 1H), 1.37 (d, J = 4.4 Hz, 9H); ^{13}C NMR (101 MHz, $CDCl_3$) δ_C 186.0, 157.8, 157.4, 136.2, 133.3, 130.3 (2C), 129.5 (2C), 128.7 (2C), 126.9, 125.4 (2C), 83.4, 40.9, 38.4, 35.2, 31.1(3C); IR νmax 3030, 1660, 1601, 1403, 1375, 860, 750, 700 cm^{-1}; HRMS (EI) *m/z* calcd for $C_{21}H_{24}NO_2$ $[M + H]^+$ 322.1802, found 322.1808. These data are consistent with reported literature values[5].

[1,1'-Biphenyl]-4-yl(5-benzyl-4,5-dihydroisoxazol-3-yl)methanone (3h).

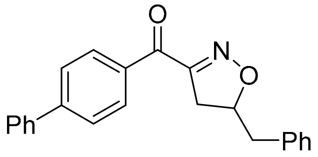

The compound was prepared following the general procedure. The title compound was isolated as a pale yellow oily liquid, yield 68%. ^{1}H NMR (400 MHz, $CDCl_3$) δ_H 8.24 – 8.20 (m, 2H), 7.70 – 7.68 (m, 1H), 7.69 – 7.68 (m, 1H), 7.65 – 7.64 (m, 1H), 7.63 – 7.62 (m, 1H), 7.50 – 7.49 (m, 1H), 7.48 – 7.47 (m, 1H), 7.46 – 7.45 (m, 1H), 7.43 – 7.42 (m, 1H), 7.35 – 7.33 (m, 1H), 7.32 (m, 1H), 7.30 – 7.28 (m, 2H), 5.08 (ddt, J = 10.8, 7.9, 6.3 Hz, 1H), 3.38 (dd, J = 17.6, 10.8 Hz, 1H), 3.18 – 3.09 (m, 2H), 2.98 (dd, J = 14.0, 6.5 Hz, 1H); ^{13}C NMR (101 MHz, $CDCl_3$) δ_C 186.0, 157.9, 146.4, 139.9, 136.2, 134.6, 131.0 (2C), 129.6 (2C), 129.1 (2C), 128.8 (2C), 128.5 (2C), 127.5 (2C), 127.1 (2C), 83.7, 41.1, 38.5; IR νmax 3029, 1655, 1648, 1401, 1362, 842, 746, 693 cm^{-1}; HRMS (EI) *m/z* calcd for $C_{23}H_{20}NO_2$ $[M + H]^+$ 342.1489, found 342.1483. These data are consistent with reported literature values[5].

(5-Benzyl-4,5-dihydroisoxazol-3-yl)(4-fluorophenyl)methanone (3i)

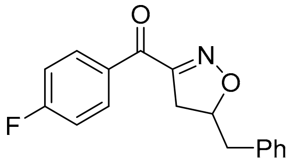

The compound was prepared following the general procedure. The title compound was isolated as a pale yellow oily liquid, yield 71%. ^{1}H NMR (400 MHz, $CDCl_3$) δ_H 8.24 – 8.15 (m, 2H), 7.37 – 7.29 (m, 2H), 7.28 – 7.25 (m, 3H), 7.15 – 7.09 (m, 2H), 5.07 (ddt, J = 10.9, 7.9, 6.3 Hz, 1H), 3.35 (dd, J = 17.6, 10.9 Hz, 1H), 3.16 – 3.06 (m, 1H), 2.97 (dd, J = 14.0, 6.5 Hz, 1H). ^{13}C NMR (101 MHz, $CDCl_3$) δ_C 184.7, 167.4, 164.9, 157.7, 136.0, 133.1, 133.1, 129.5 (2C), 128.7 (2C), 127.0, 115.7, 115. 5, 83.6, 40.9, 38.3. IR νmax 3032, 2991, 2463, 2465, 1747, 1734, 1683, 1244, 1236, 1166, 987, 846, 748, 713, 626 cm^{-1}; HRMS (EI) *m/z* calcd for $C_{17}H_{15}NO_2F$ $[M + H]^+$ 284.1087, found 284.1089.

(5-Octyl-4,5-dihydroisoxazol-3-yl)(phenyl)methanone (5a)

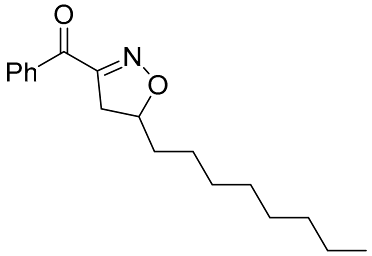

The compound was prepared following the general procedure. The title compound was isolated as a pale yellow oily liquid, yield 86%. ^{1}H NMR (400 MHz, $CDCl_3$) δ_H 8.22 – 8.17 (m, 2H), 7.61 – 7.55 (m, 1H), 7.46 (m, 2H), 4.79 (ddt, J = 10.9, 8.4, 6.6 Hz, 1H), 3.39 (dd, J = 17.4, 10.9 Hz, 1H), 3.00 (dd, J = 17.4, 8.5 Hz, 1H), 1.85 – 1.73 (m, 1H), 1.70 – 1.58 (m, 1H), 1.38 – 1.22 (m, 12H), 0.91 – 0.85 (m, 3H); ^{13}C NMR (101 MHz, $CDCl_3$) δ_C 186.7, 157.9, 136.0, 133.6, 130.5 (2C), 128.5 (2C), 83.7, 38.9, 35.3, 31.9, 29.6, 29.5, 29.3, 25.4, 22.8, 14.2; IR νmax 3062, 2948, 1635, 1541, 760, 710 cm^{-1}; HRMS (EI) *m/z* calcd for $C_{18}H_{26}NO_2$ $[M + H]^+$ 288.1958, found 288.1961. These data are consistent with reported literature values[4,5].

(5-(2-Methylbenzyl)-4,5-dihydroisoxazol-3-yl)(phenyl)methanone (5b)

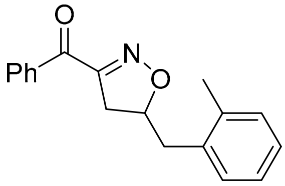

The compounds was prepared following the general procedure as isomers. The title compound was isolated as a pale yellow oily liquid, yield 87%. ^{1}H NMR (400 MHz, $CDCl_3$) δ_H 8.24 – 8.19 (m, 2H), 7.63 – 7.58 (m, 1H), 7.51 – 7.45 (m, 2H), 7.23 – 7.18 (m, 4H), 5.11 – 5.04 (m, 1H), 3.37 (ddd, J = 17.5, 10.7, 0.9 Hz, 1H), 3.22 – 3.12 (m, 2H), 2.94 (ddd, J = 14.3, 6.7, 1.2 Hz, 1H), 2.39 (s, 3H); ^{13}C NMR (101 MHz, $CDCl_3$) δ_C 186.5, 157.9, 136.6, 135.9, 134.6, 133.6, 130.6, 130.4 (2C), 130.0, 128.4 (2C), 127.1, 126.3, 82.9, 38.6, 38.1, 19.8; IR νmax 3028, 2940, 1660, 1570, 750, 690 cm^{-1}; HRMS (EI) *m/z* calcd for $C_{18}H_{18}NO_2$ $[M + H]^+$ 280.1332, found 280.1335. These data are consistent with reported literature values[5].

(5-(3-Methylbenzyl)-4,5-dihydroisoxazol-3-yl)(phenyl)methanone (5c)

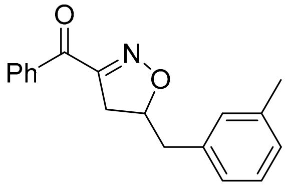

The compound was prepared following the general procedure. The title compound was isolated as a pale yellow oily liquid, yield 85%. ^{1}H NMR (400 MHz, $CDCl_3$) δ_H 8.15 (ddd, J = 8.5, 2.8, 1.3 Hz, 2H), 7.65 – 7.59 (m, 1H), 7.49 – 7.43 (m, 2H), 7.29 – 7.20 (m, 1H), 7.11 – 7.04 (m, 3H), 5.07 (ddt, J = 10.9, 8.1, 4.2 Hz, 1H) , 3.35 (ddd, J = 17.5, 10.8, 2.3 Hz, 1H), 3.17 – 3.06 (m, 2H), 2.92 (ddd, J = 14.1, 6.7, 2.4 Hz, 1H), 2.36 (s, 3H); ^{13}C NMR (101 MHz, $CDCl_3$) δ_C 186.6, 157.8, 138.4, 136.1, 135.9, 133.7, 130.4 (2C), 130.4, 128.7, 128.5 (2C), 127.9, 126.6, 83.8, 40.9, 38.5, 21.5; IR νmax 3048, 2908, 1661, 1581, 862, 750, 691 cm^{-1}; HRMS (EI) *m/z* calcd for $C_{18}H_{18}NO_2$ $[M + H]^+$ 280.1332, found 280.1340. These data are consistent with reported literature values[5].

(5-(4-Methylbenzyl)-4,5-dihydroisoxazol-3-yl)(phenyl)methanone (5d)

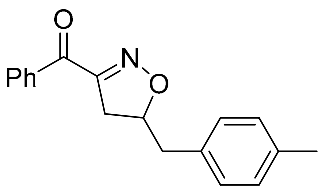

The compound was prepared following the general procedure. The title compound was isolated as a pale yellow oily liquid, yield 85%. ^{1}H NMR (400 MHz, $CDCl_3$) δ_H 8.13 – 8.10 (m, 2H), 7.59 (tt, J = 7.0, 1.3 Hz, 1H) , 7.48 – 7.43 (m, 2H), 7.17 – 7.11 (m, 4H), 5.08 – 5.00 (m, 1H), 3.34 (dd, J = 17.6, 10.8 Hz, 1H), 3.14 – 3.03 (m, 2H), 2.92 (dd, J = 14.0, 6.6 Hz, 1H), 2.33 (s, 3H); ^{13}C NMR (101 MHz, $CDCl_3$) δ_C 186.5, 157.7, 136.6, 135.9, 133.5, 132.9, 130.3 (2C), 129.4 (4C), 128.4 (2C), 83.7, 40.5, 38.3, 21.1; IR νmax 3039, 2909, 1710, 1609, 822, 741, 680 cm^{-1}; HRMS (EI) *m/z* calcd for $C_{18}H_{18}NO_2$ $[M + H]^+$ 280.1332, found 280.1339. These data are consistent with reported literature values[5].

(5-(4-Methoxybenzyl)-4,5-dihydroisoxazol-3-yl)(phenyl)methanone (5e)

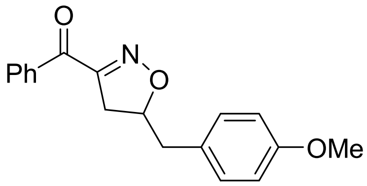

The compound was prepared following the general procedure. The title compound was isolated as a pale yellow oily liquid, yield 89%. ^{1}H NMR (400 MHz, $CDCl_3$) δ_H 8.12 (m, 2H), 7.58 (m, 1H), 7.45 (m, 2H), 7.18 (m, 2H), 6.86 (m, 2H), 5.08 – 4.96 (m, 1H), 3.78 (s, 3H), 3.34 (dd, J = 17.6, 10.8 Hz, 1H), 3.14 – 2.99 (m, 2H), 2.91 (dd, J = 14.1, 6.4 Hz, 1H); ^{13}C NMR (101 MHz, $CDCl_3$) δ_C 186.6, 158.7, 157.8, 135.9, 133.6, 130.6 (2C), 130.4 (2C), 128.4 (2C), 128.1, 114.2 (2C), 83.8, 55.3, 40.0, 38.3; IR νmax 3050, 2850, 1670, 1580, 820, 750, 691 cm^{-1}; HRMS (EI) *m/z* calcd for $C_{18}H_{18}NO_3$ $[M + H]^+$ 296.1281, found 296.1285. These data are consistent with reported literature values[5].

(3a,4,5,6,7,7a-Hexahydrobenzo[d]isoxazol-3-yl)(phenyl)methanone (5f)

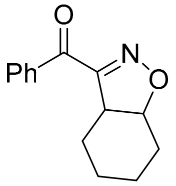

The compound was prepared following the general procedure. The title compound was isolated as a pale yellow oily liquid, yield 69%. ^{1}H NMR (400 MHz, $CDCl_3$) δ_H 8.24 – 8.17 (m, 2H), 7.66 – 7.57 (m, 1H), 7.54 – 7.44 (m, 2H), 4.61 (dt, J = 7.9, 3.9 Hz, 1H), 3.49 – 3.39 (m, 1H), 2.28 – 2.17 (m, 1H), 2.14 – 2.03 (m, 1H), 1.91– 1.77 (m, 1H), 1.68 – 1.54 (m, 3H), 1.37 – 1.27 (m, 2H); ^{13}C NMR (101 MHz, $CDCl_3$) δ_C 187.0, 163.8, 136.4, 133.6, 130.4 (2C), 128.5 (2C), 82.3, 44.3, 25.6, 25.0, 21.7, 19.9; IR vmax 3060, 2940, 1660, 1550, 747, 704 cm^{-1}; HRMS (EI) *m/z* calcd for $C_{14}H_{16}NO_2$ $[M + H]^+$ 230.1176, found 230.1182. These data are consistent with reported literature values[4,5].

(5-Benzyl-4,5-dihydroisoxazol-3-yl)ethanone (7a)

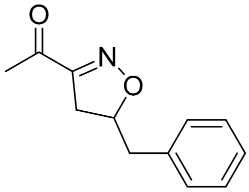

The compound was prepared following the general procedure. The title compound was isolated as a pale yellow oily liquid, yield 23%. ^{1}H NMR (400 MHz, $CDCl_3$) δ_H 7.36 – 7.28 (m, 2H), 7.25 – 7.19 (m, 3H), 5.10 – 4.97 (m, 1H), 3.15 – 3.04 (m, 2H), 2.92 – 2.82 (m, 2H), 2.48 – 2.45 (s, 3H); ^{13}C NMR (101 MHz, $CDCl_3$) δ_C 193.23, 158.19, 135.95, 129.41 (2C), 128.70 (2C), 127.00, 84.93, 40.89, 36.25, 26.60; IR vmax 2978, 1687, 1244, 925, 790, 700 cm^{-1}; These data are consistent with reported literature values[6].

(5-Benzyl-4,5-dihydroisoxazol-3-yl)isobutanone (7b)

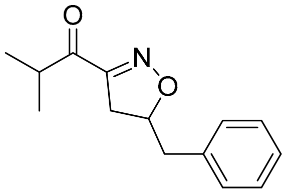

The compound was prepared following the general procedure. The title compound was isolated as a pale yellow oily liquid, yield 20%.^{1}H NMR (400 MHz, , $CDCl_3$) δ_H 7.33

– 7.28 (t, J = 7.2 Hz, 2H), 7.25 – 7.20 (m, 3H), 5.07 – 4.98 (m, 1H), 3.51 – 3.37 (m, 1H), 3.13 – 3.02 (m, 2H), 2.94 – 2.78 (m, 2H), 1.18 – 1.04 (dd, J = 6.9, 15.2 Hz, 6H); ^{13}C NMR (101 MHz, $CDCl_3$) δ_C 199.69, 156.68, 135.97, 129.45 (2C), 128.66 (2C), 126.96, 84.28, 40.89, 37.01, 36.70, 18.62, 18.54; IR νmax 2931, 1685, 1172, 927, 916, 896, 700 cm^{-1}; HRMS (ESI) m/z calcd for $C_{14}H_{21}N_2O_2$ [M + NH_4]$^+$ 249.1600, found 249.1603.

Ethyl 3-benzoyl-4,5-dihydroisoxazole-5-carboxylate (9a)

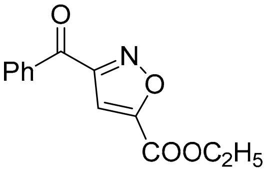

The compound was prepared following the general procedure. The title compound was isolated as a pale yellow oily liquid, yield 85%. ^{1}H NMR (400 MHz, $CDCl_3$) δ_H 8.33 – 8.28 (m, 2H), 7.71 – 7.65 (m, 1H), 7.59 – 7.51 (m, 2H), 7.43 (s, 1H), 4.48 (q, J = 7.1 Hz, 2H), 1.44 (t, J = 7.1 Hz, 3H); ^{13}C NMR (101 MHz, $CDCl_3$) δ_C 184.7, 162.3, 161.3, 156.4, 135.3, 134.6, 130.9 (2C), 128.9 (2C), 110.2, 62.8, 14.3; IR νmax 3058, 2945, 1655, 1545, 740, 702 cm^{-1}; HRMS (EI) *m/z* calcd for $C_{13}H_{14}NO_2$ [M + H]$^+$ 248.0917, found 248.0912. These data are consistent with reported literature values[4,5].

(5-Butylisoxazol-3-yl)(phenyl)methanone (9b).

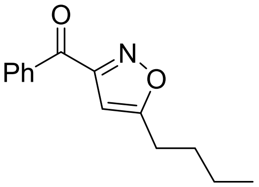

The compound was prepared following the general procedure. The title compound was isolated as a pale yellow oily liquid, yield 88%. ^{1}H NMR (400 MHz, $CDCl_3$) δ_H 8.33 – 8.25 (m, 2H), 7.66 – 7.57 (m, 1H), 7.53 – 7.46 (m, 2H), 6.51 (t, J = 0.8 Hz, 1H), 2.82 (t, J = 7.6 Hz, 2H), 1.73 (dt, J = 15.2, 7.5 Hz, 2H), 1.42 (dq, J = 14.6, 7.4 Hz, 2H), 0.95 (t, J = 7.4 Hz, 3H); ^{13}C NMR (101 MHz, $CDCl_3$) δ_C 186.2, 174.8, 161.9, 135.9, 133.9, 130.7 (2C), 128.6 (2C), 101.7, 29.6, 26.4, 22.3, 13.8. IR νmax 3075, 2950, 2875, 1670, 1590, 740, 690 cm^{-1}; HRMS (EI) *m/z* calcd for $C_{14}H_{16}NO_2$ [M + H]$^+$

230.1176, found 230.1171. These data are consistent with reported literature values[4,5].

References

1. Lee, J. J.; Kim, J.; Jun, Y. M.; Lee, B. M.; Kim, B. H., Indium-mediated one-pot synthesis of benzoxazoles or oxazoles from 2-nitrophenols or 1-aryl-2-nitroethanones. *Tetrahedron* **2009,** *65* (43), 8821-8831.
2. Zhang, H. Q.; Pan, X. H.; Zhang, K.; Wang, H. Y.; Wang, J, H., Synthesis of *α*-nitro ketones. *Journal of Shihezi University (Natural Science Edition)* **2017,** *35* (05), 602-605.
3. Lindsay, A. C.; Kilmartin, P. A.; Organic, J. S. J.; chemistry, b., Synthesis of 3-nitroindoles by sequential paired electrolysis. **2021,** *19* (36), 7903-7913.
4. Itoh, K.-i.; Aoyama, T.; Satoh, H.; Fujii, Y.; Sakamaki, H.; Takido, T.; Kodomari, M., Application of silica gel-supported polyphosphoric acid (PPA/SiO_2) as a reusable solid acid catalyst to the synthesis of 3-benzoylisoxazoles and isoxazolines. *Tetrahedron Letters* **2011,** *52* (51), 6892-6895.
5. Pan, X. H.; Xin, X. B.; Mao, Y.; Li, X.; Zhao, Y, N.; Liu, Y, D.; Zhang, K.; Yang, X, D.; Wang, J, H., 3-Benzoylisoxazolines by 1,3-Dipolar Cycloaddition: Chloramine-T-Catalyzed Condensation of *α*-Nitroketones with Dipolarophiles. *Molecules* **2021,** *26*(12), 3491.
6. Dai, P.; Tan, X.; Luo, Q.; Yu, X.; Zhang, S. G; Liu, F.; Zhang, H. W., Synthesis of 3-Acyl-isoxazoles and Δ2-Isoxazolines from Methyl Ketones, Alkynes or Alkenes, and tert-Butyl Nitrite via a Csp^3-H Radical Functionalization/Cycloaddition Cascade. *Organic letters* **2019,** *21* (13), 5096-5100.

^{1}H and ^{13}C NMR spectra

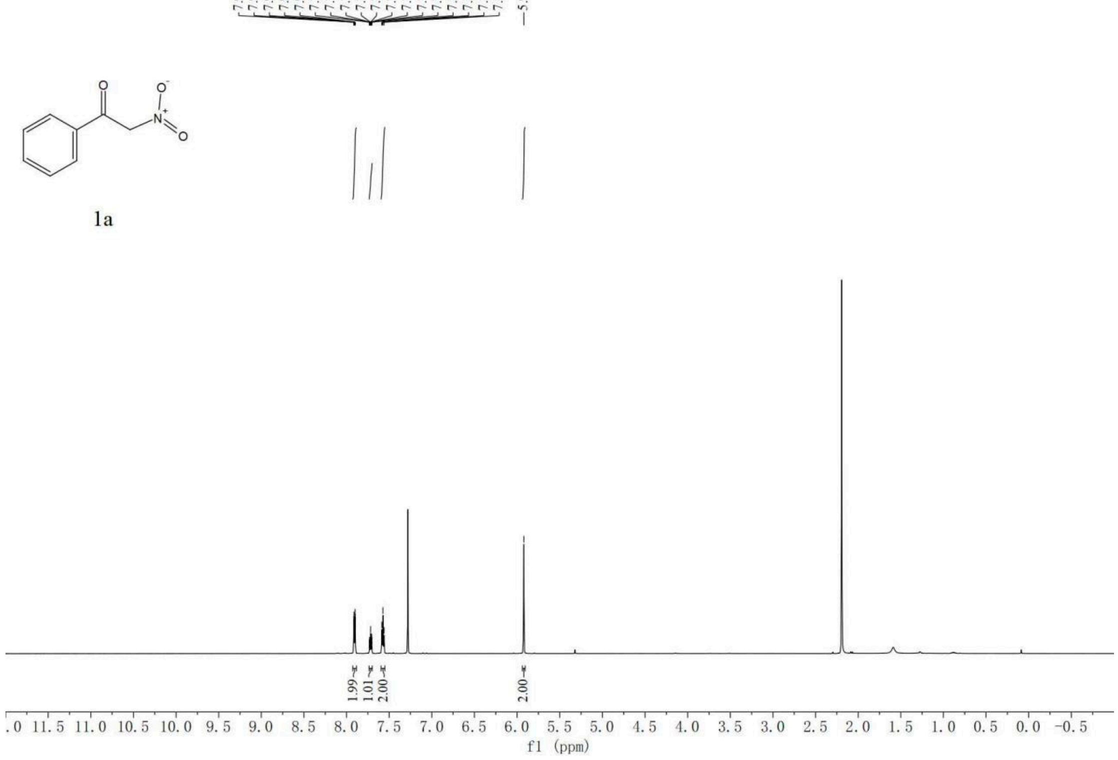

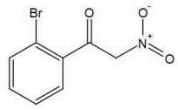

1b

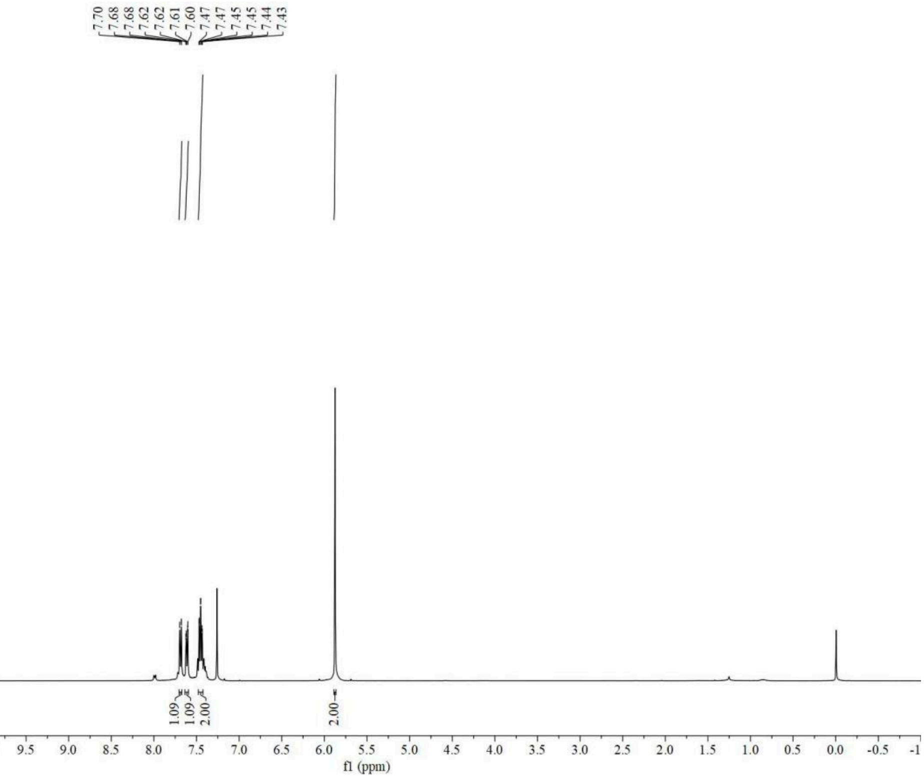

yk-br
7.70
7.68
7.68
7.62
7.62
7.61
7.60
7.47
7.47
7.45
7.45
7.44
7.43
1.09
1.09
2.00
2.00
.0 11.5 11.0 10.5 10.0 9.5 9.0 8.5 8.0 7.5 7.0 6.5 6.0 5.5 5.0 4.5 4.0 3.5 3.0 2.5 2.0 1.5 1.0 0.5 0.0 -0.5 -1
f1 (ppm)

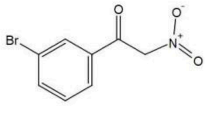

1c

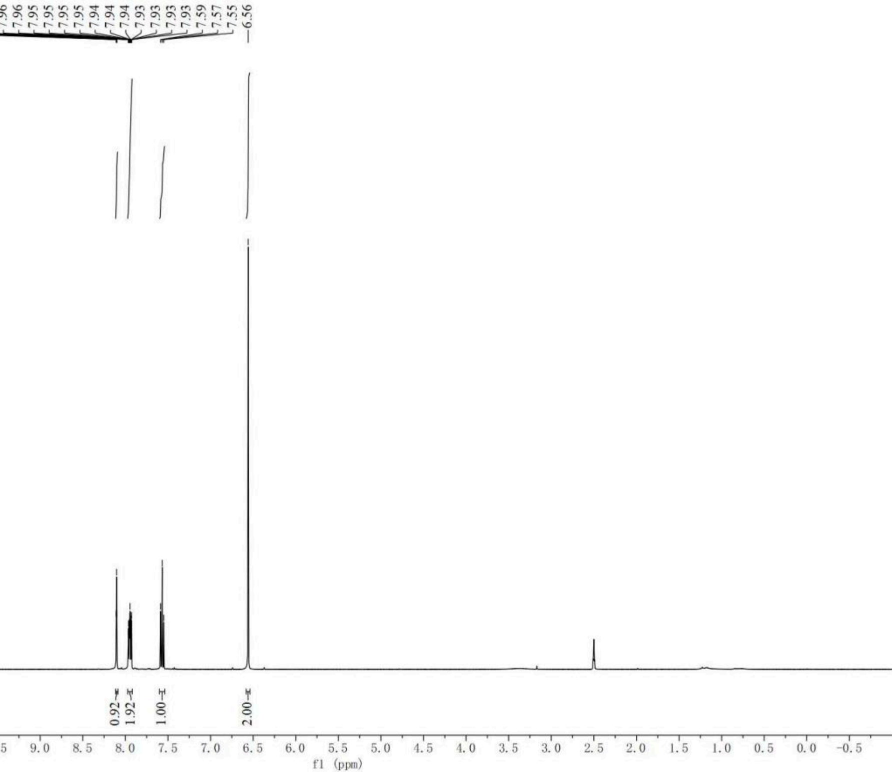

yezyl-jbr
8.11
8.10
8.10
7.97
7.96
7.96
7.95
7.95
7.95
7.95
7.94
7.94
7.94
7.93
7.93
7.93
7.59
7.57
7.55
6.56
0.92
1.92
1.00
2.00
.0 11.5 11.0 10.5 10.0 9.5 9.0 8.5 8.0 7.5 7.0 6.5 6.0 5.5 5.0 4.5 4.0 3.5 3.0 2.5 2.0 1.5 1.0 0.5 0.0 -0.5
f1 (ppm)

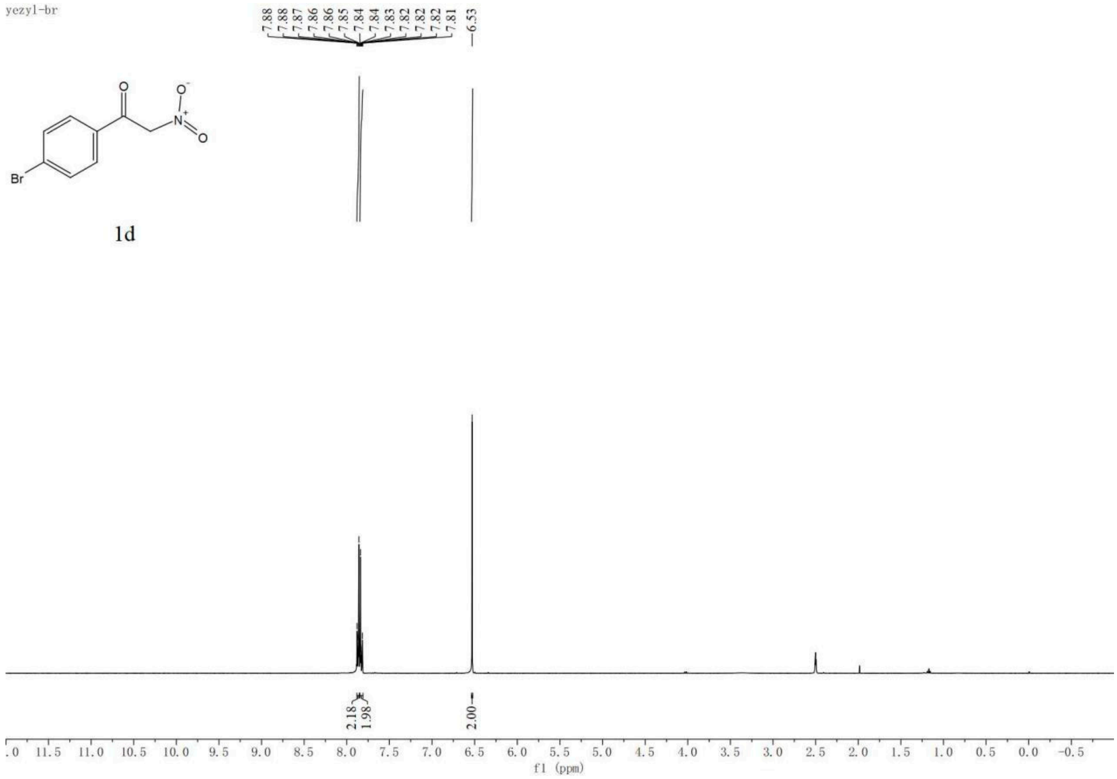
yezyl-br
7.88
7.88
7.87
7.86
7.86
7.85
7.84
7.84
7.83
7.82
7.82
7.82
7.81
6.53
Br
1d
2.18
1.98
2.00
f1 (ppm)

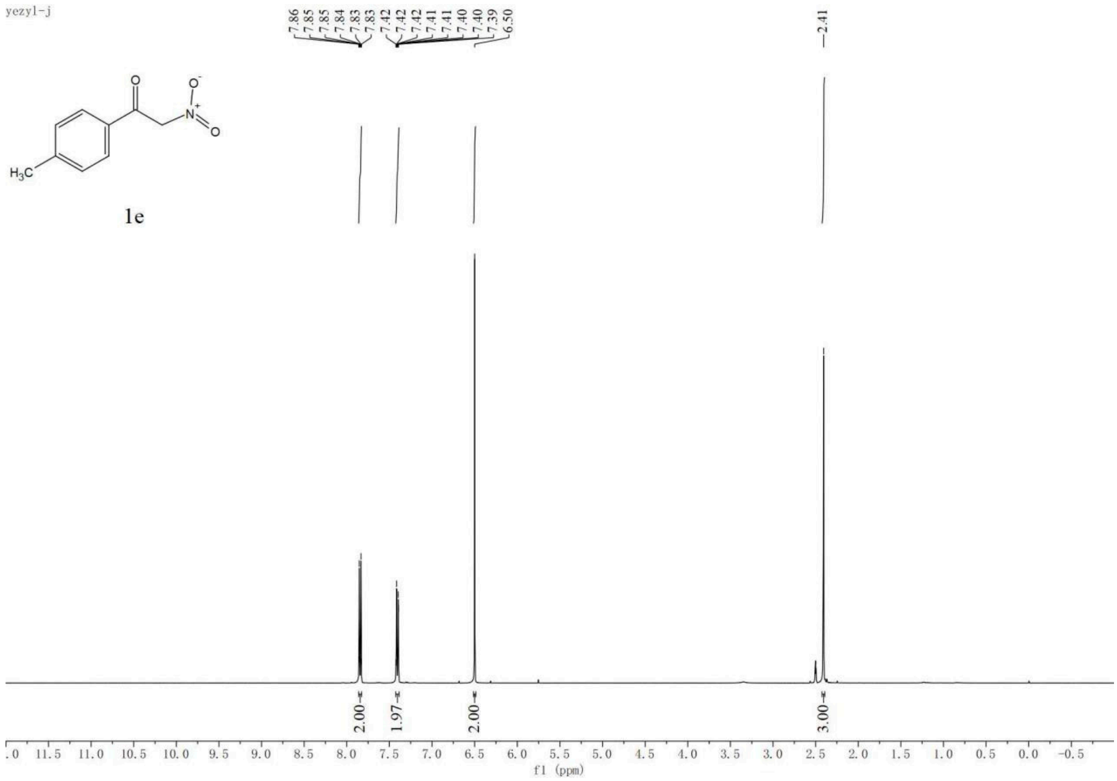
yezyl-j
7.86
7.85
7.85
7.84
7.83
7.83
7.42
7.42
7.42
7.41
7.41
7.40
7.40
7.39
6.50
2.41
H_3C
1e
2.00
1.97
2.00
3.00
f1 (ppm)

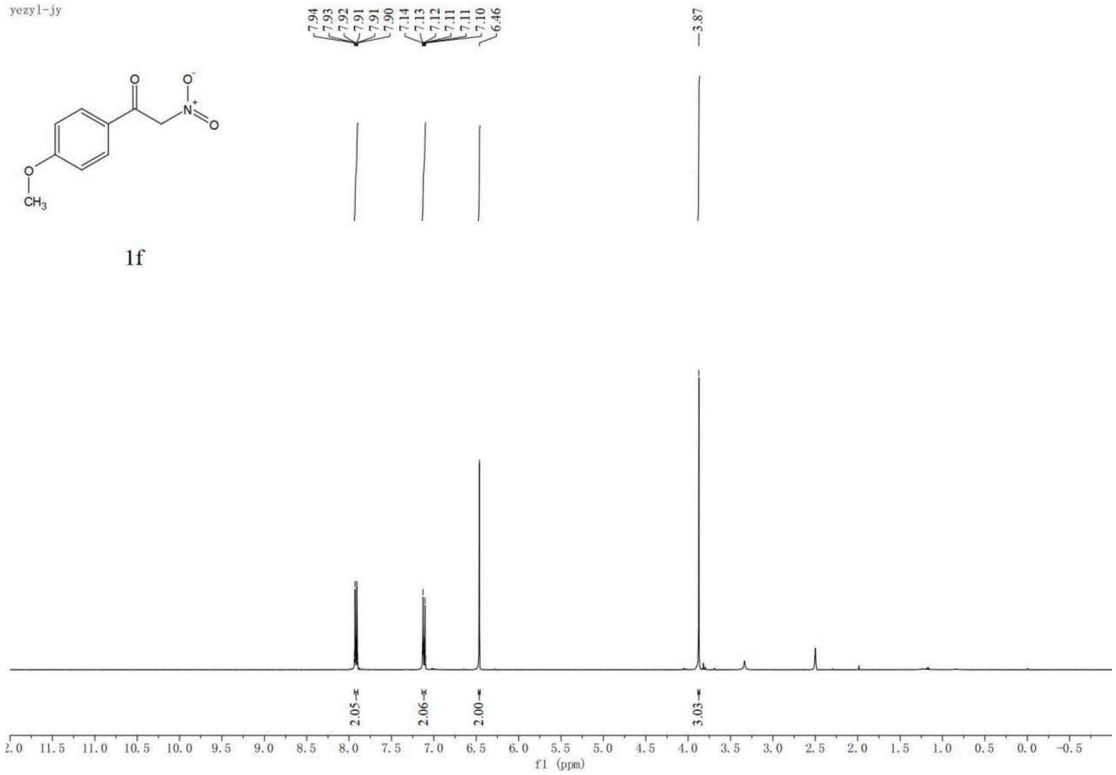
yezyl-jy
7.94
7.93
7.92
7.91
7.91
7.90
7.14
7.13
7.12
7.11
7.11
7.10
6.46
3.87
1f
2.05
2.06
2.00
3.03
f1 (ppm)

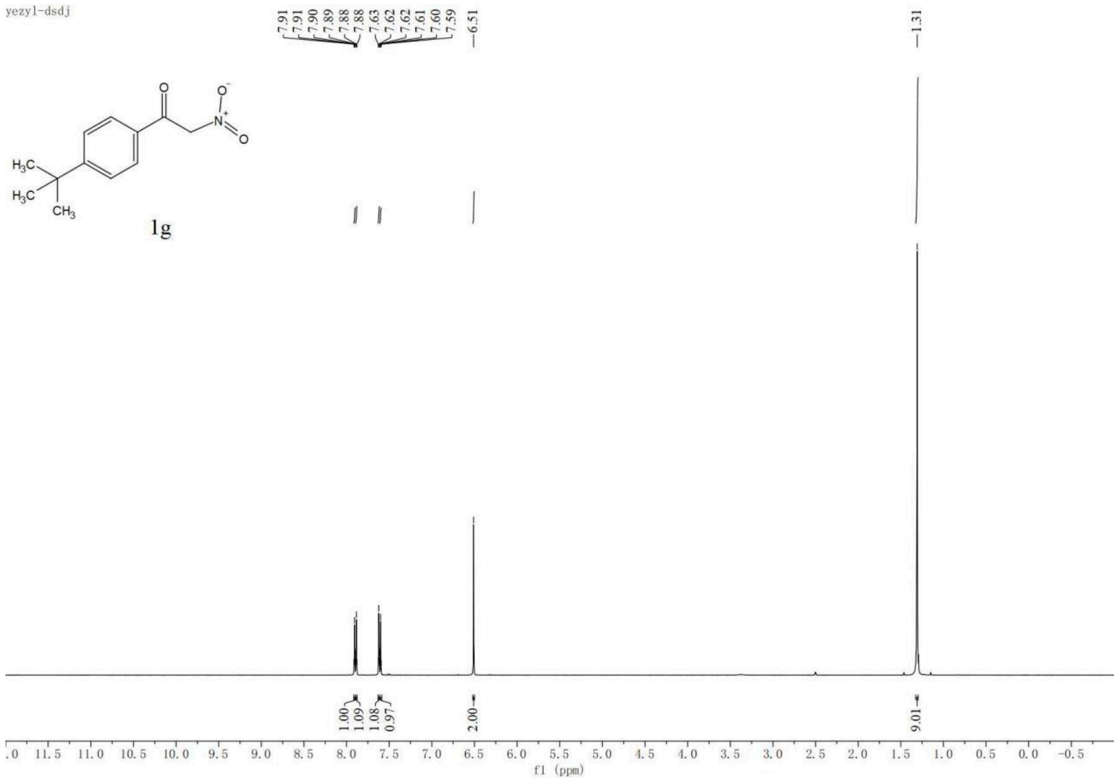
yezyl-dsdj
7.91
7.91
7.90
7.89
7.88
7.88
7.63
7.62
7.62
7.61
7.60
7.59
6.51
1.31
1g
1.00
1.09
1.08
0.97
2.00
9.01
f1 (ppm)

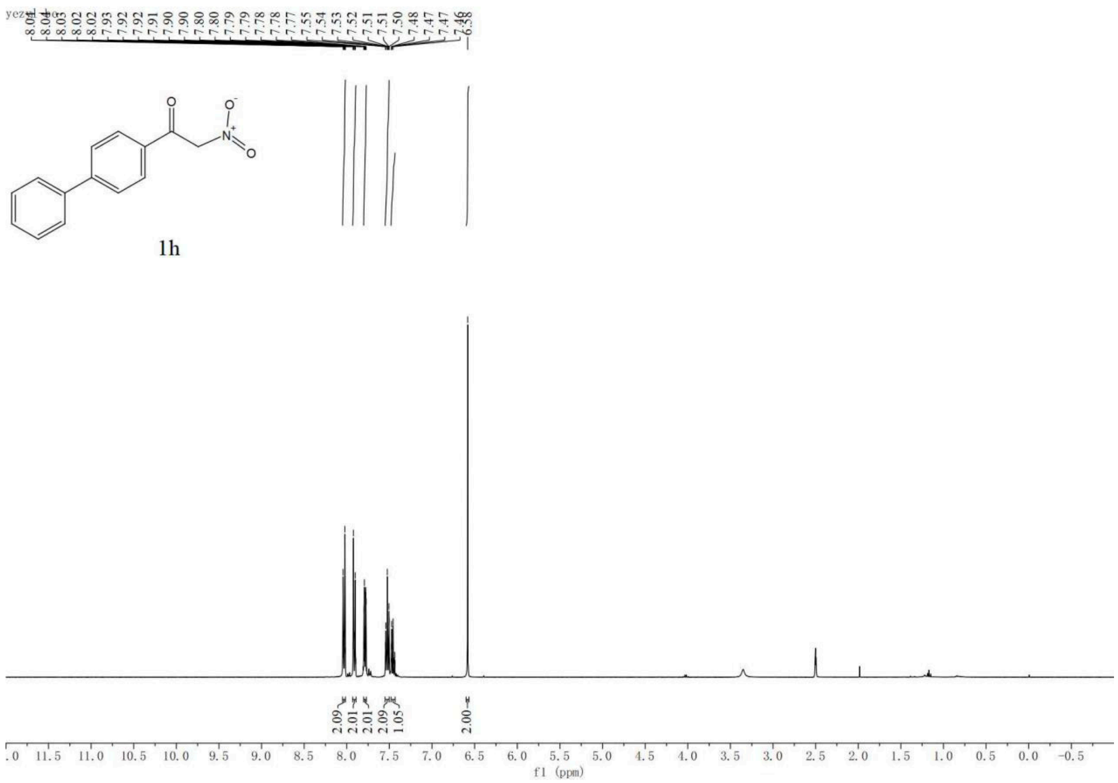
1h
f1 (ppm)

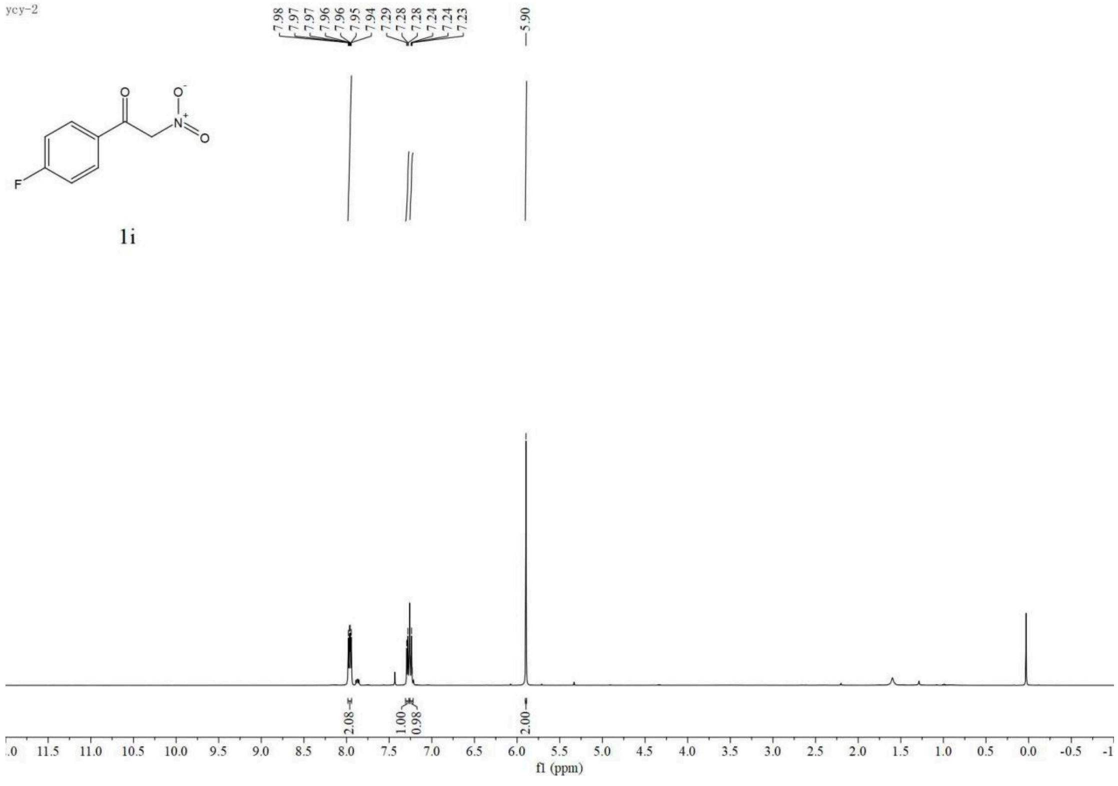
ycy-2
1i
f1 (ppm)

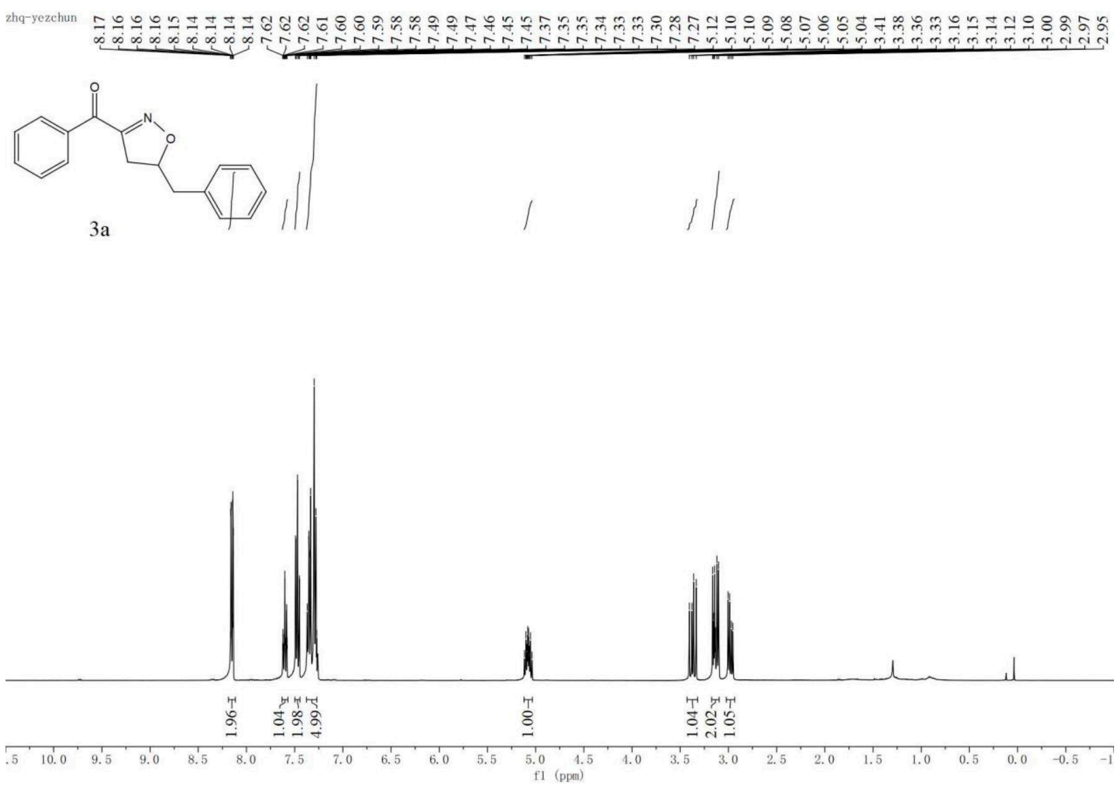
zhq-yezchun
8.17 8.16 8.16 8.16 8.15 8.14 8.14 8.14 8.14 7.62 7.62 7.62 7.61 7.60 7.59 7.58 7.58 7.49 7.49 7.47 7.46 7.45 7.45 7.37 7.35 7.35 7.34 7.33 7.33 7.30 7.28 7.27 5.12 5.10 5.10 5.09 5.08 5.07 5.06 5.05 5.04 3.41 3.38 3.36 3.33 3.16 3.15 3.14 3.12 3.10 3.00 2.99 2.97 2.95
3a
1.96
1.04
1.98
4.99
1.00
1.04
2.02
1.05
f1 (ppm)

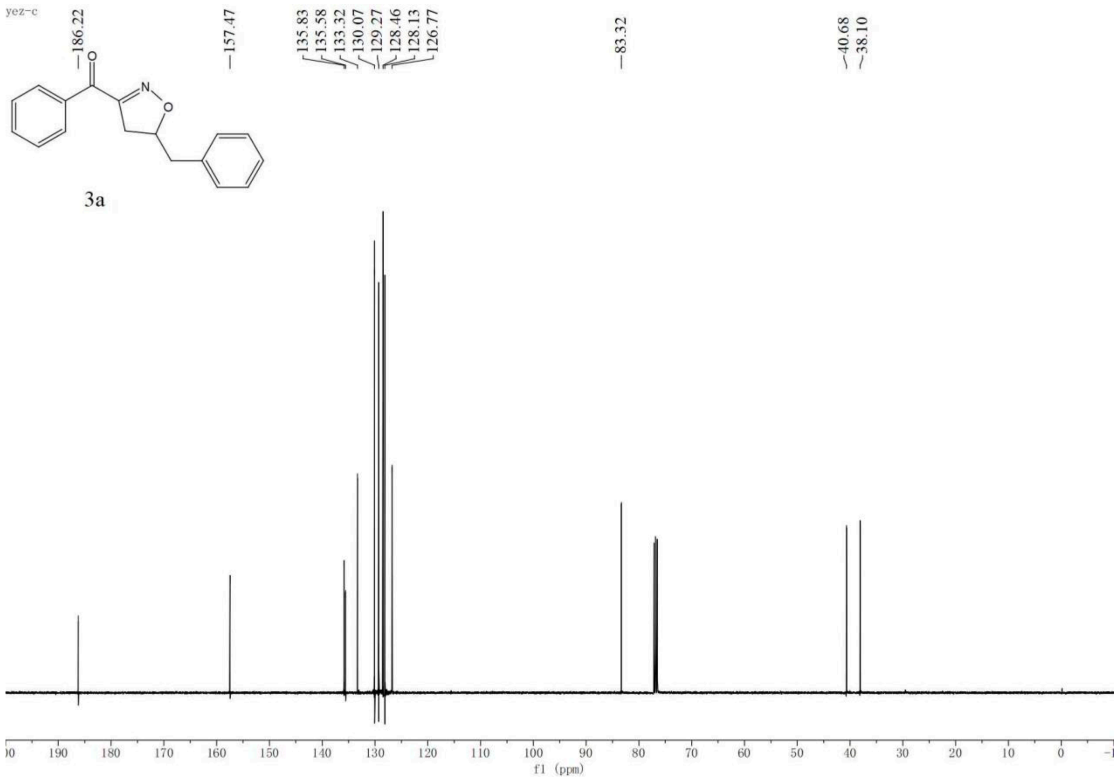
yez-c
186.22
157.47
135.83 135.58 133.32 130.07 129.27 128.46 128.13 126.77
83.32
40.68 38.10
3a
f1 (ppm)

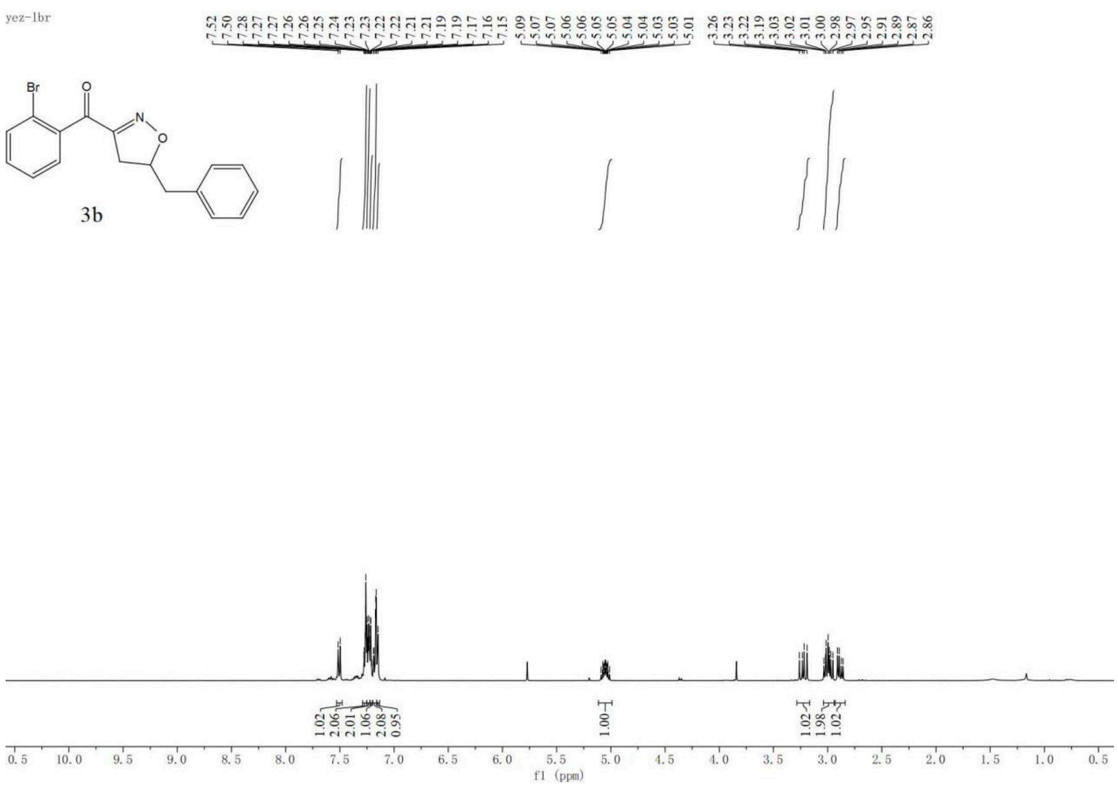
yez-1br
7.52 7.50 7.28 7.27 7.26 7.25 7.24 7.23 7.23 7.22 7.22 7.21 7.21 7.19 7.17 7.16 7.15
5.09 5.07 5.06 5.06 5.05 5.05 5.04 5.04 5.03 5.03 5.01
3.26 3.23 3.22 3.19 3.03 3.02 3.01 3.00 2.98 2.97 2.95 2.91 2.89 2.87 2.86
Br
O
N
O
3b
1.02 2.06 2.01 1.06 2.08 0.95
1.00
1.02 1.98 1.02
f1 (ppm)

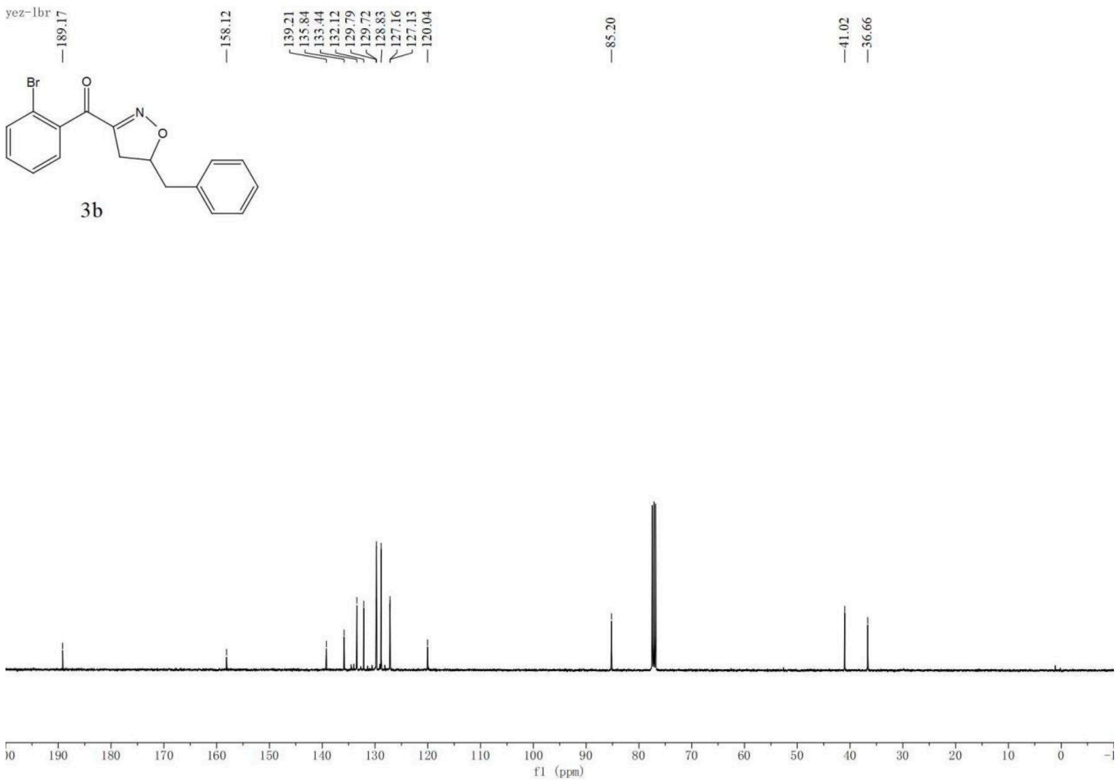
yez-1br
189.17
158.12
139.21 135.84 133.44 132.12 129.79 129.72 128.83 127.16 127.13 120.04
85.20
41.02
36.66
Br
O
N
O
3b
f1 (ppm)

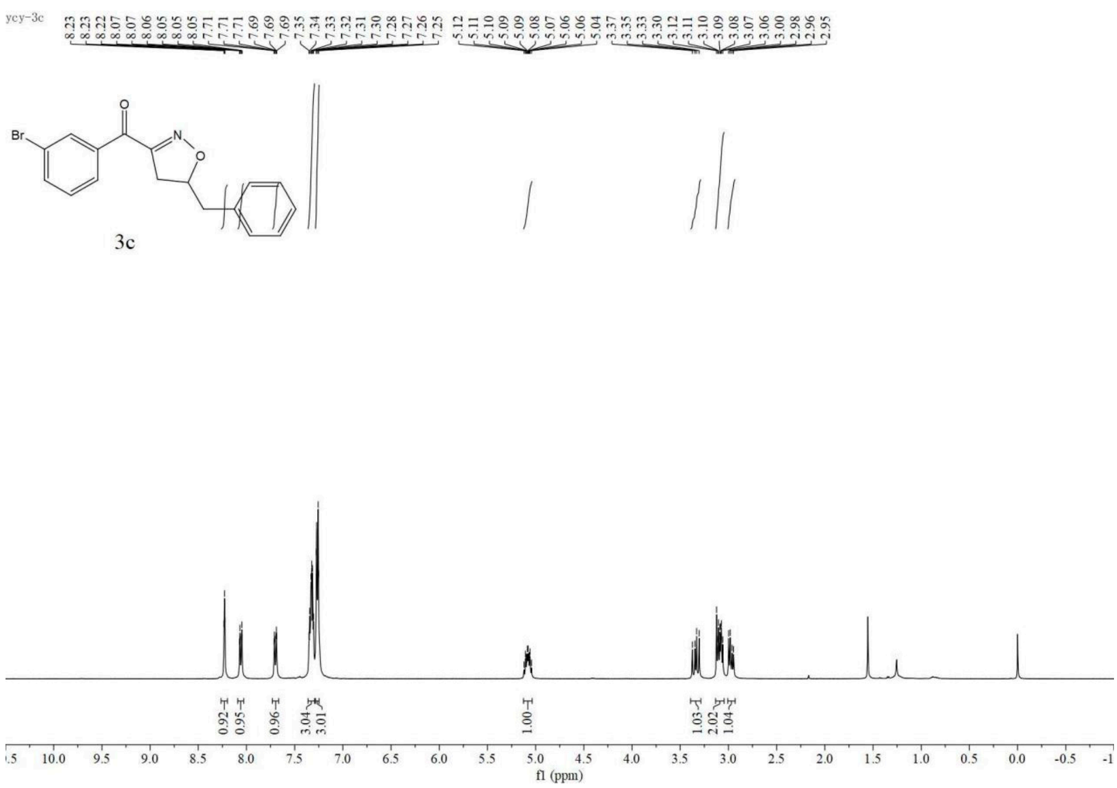
ycy-3c
8.23 8.23 8.22 8.07 8.07 8.06 8.05 8.05 8.05 7.71 7.71 7.71 7.69 7.69 7.69 7.35 7.34 7.33 7.32 7.31 7.30 7.28 7.27 7.26 7.25
5.12 5.11 5.10 5.09 5.09 5.08 5.07 5.06 5.06 5.04
3.37 3.35 3.33 3.30 3.12 3.11 3.10 3.09 3.08 3.07 3.06 3.00 2.98 2.96 2.95
Br
O
N
3c
0.92
0.95
0.96
3.04
3.01
1.00
1.03
2.02
1.04
f1 (ppm)

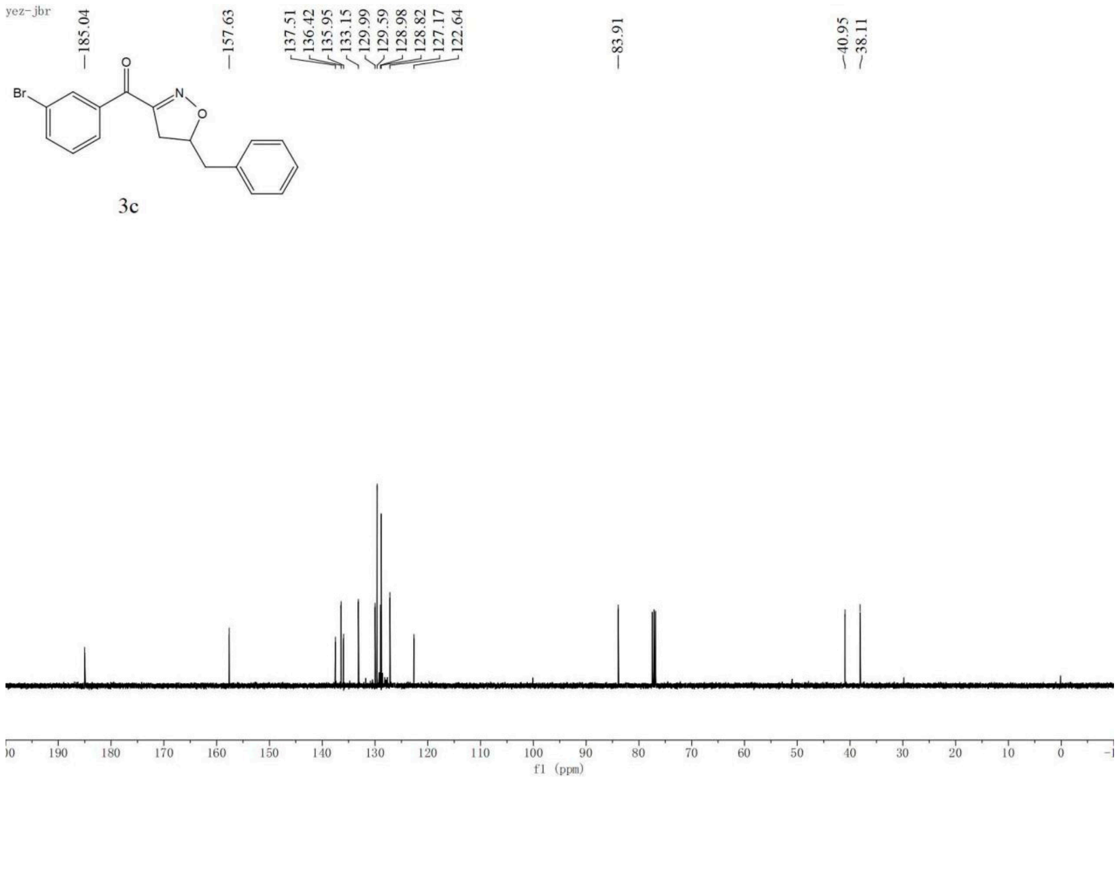
yez-jbr
185.04
157.63
137.51 136.42 135.95 133.15 129.99 129.59 128.98 128.82 127.17 122.64
83.91
40.95
38.11
Br
O
N
3c
f1 (ppm)

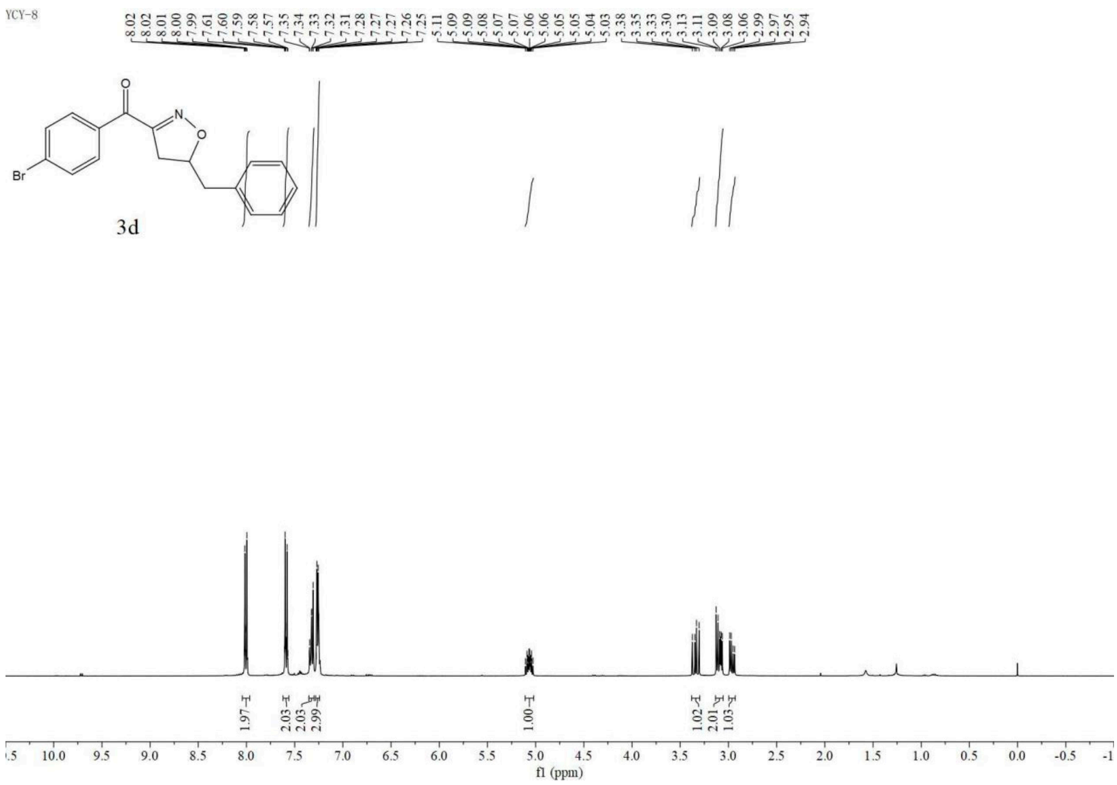
YCY-8
8.02 8.02 8.01 8.00 7.99 7.61 7.60 7.59 7.58 7.57 7.35 7.34 7.33 7.32 7.31 7.28 7.27 7.27 7.26 7.25 5.11 5.09 5.09 5.08 5.07 5.07 5.06 5.06 5.05 5.05 5.04 5.03 3.38 3.35 3.33 3.30 3.13 3.11 3.09 3.08 3.06 2.99 2.97 2.95 2.94
Br
O
N
1.97
2.03
2.03
2.99
1.00
1.02
2.01
1.03
fl (ppm)

3d

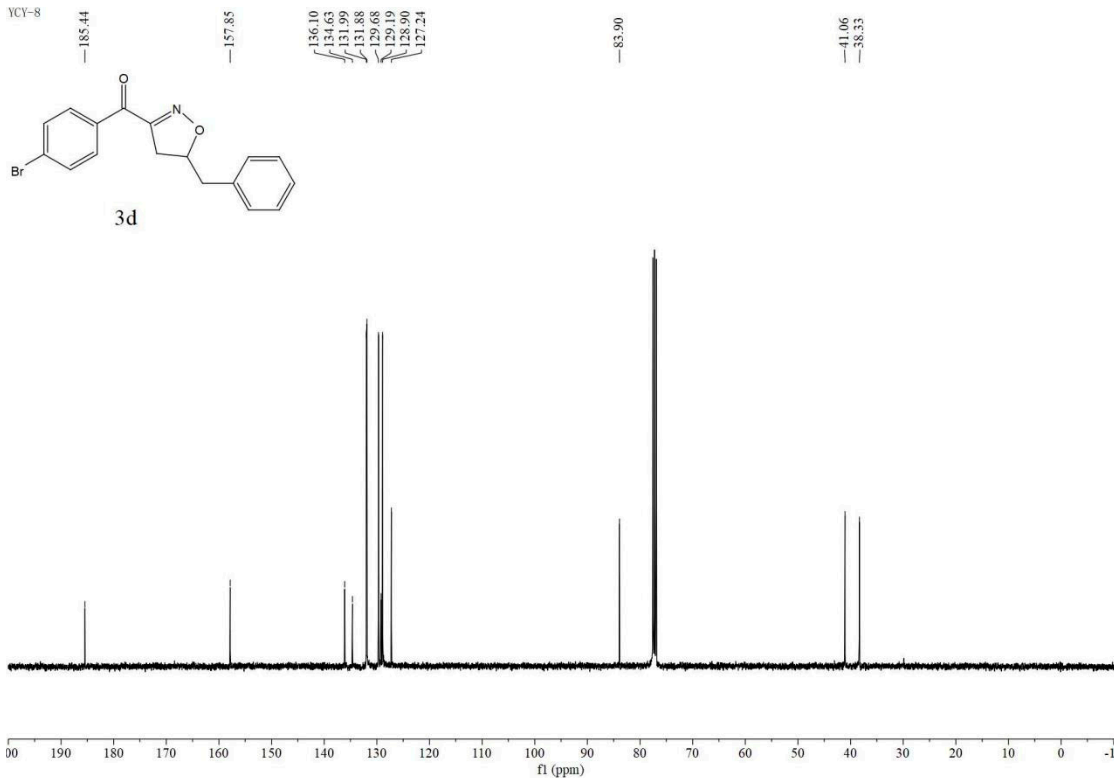
YCY-8
185.44
157.85
136.10 134.63 131.99 131.88 129.68 129.19 128.90 127.24
83.90
41.06 38.33
Br
O
N
fl (ppm)

3d

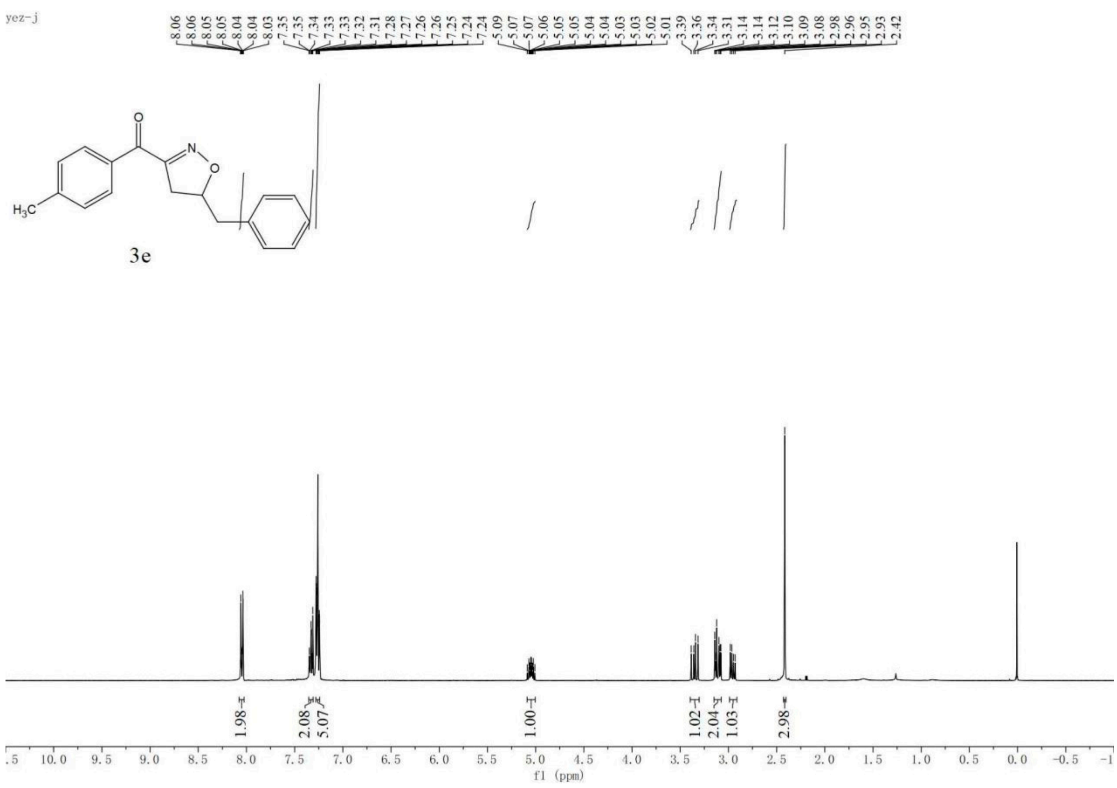
yez-j
8.06 8.06 8.05 8.05 8.04 8.04 8.03 7.35 7.35 7.34 7.33 7.33 7.32 7.31 7.28 7.27 7.26 7.26 7.25 7.24 7.24 5.09 5.07 5.07 5.06 5.05 5.05 5.04 5.04 5.03 5.03 5.02 5.01 3.39 3.36 3.34 3.31 3.14 3.14 3.12 3.10 3.09 3.08 2.98 2.96 2.95 2.93 2.42
O
N
O
H_3C
3e
1.98
2.08
5.07
1.00
1.02
2.04
1.03
2.98
f1 (ppm)

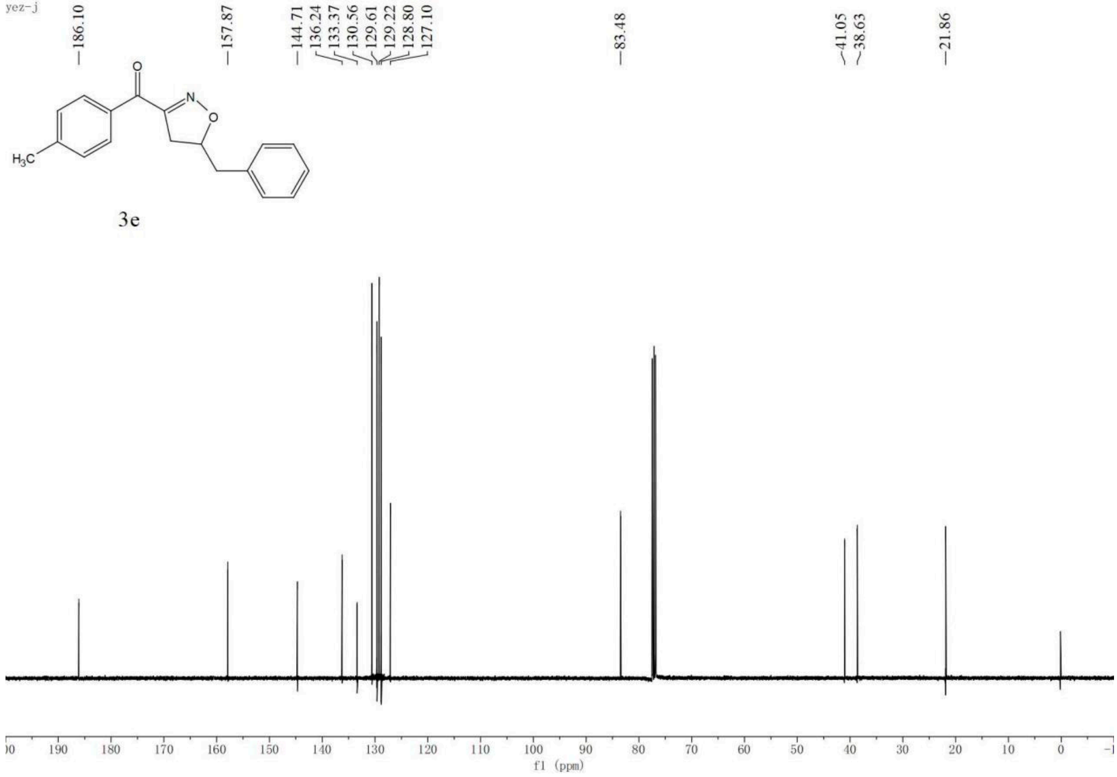
yez-j
186.10
157.87
144.71 136.24 133.37 130.56 129.61 129.22 128.80 127.10
83.48
41.05
38.63
21.86
O
N
O
H_3C
3e
f1 (ppm)

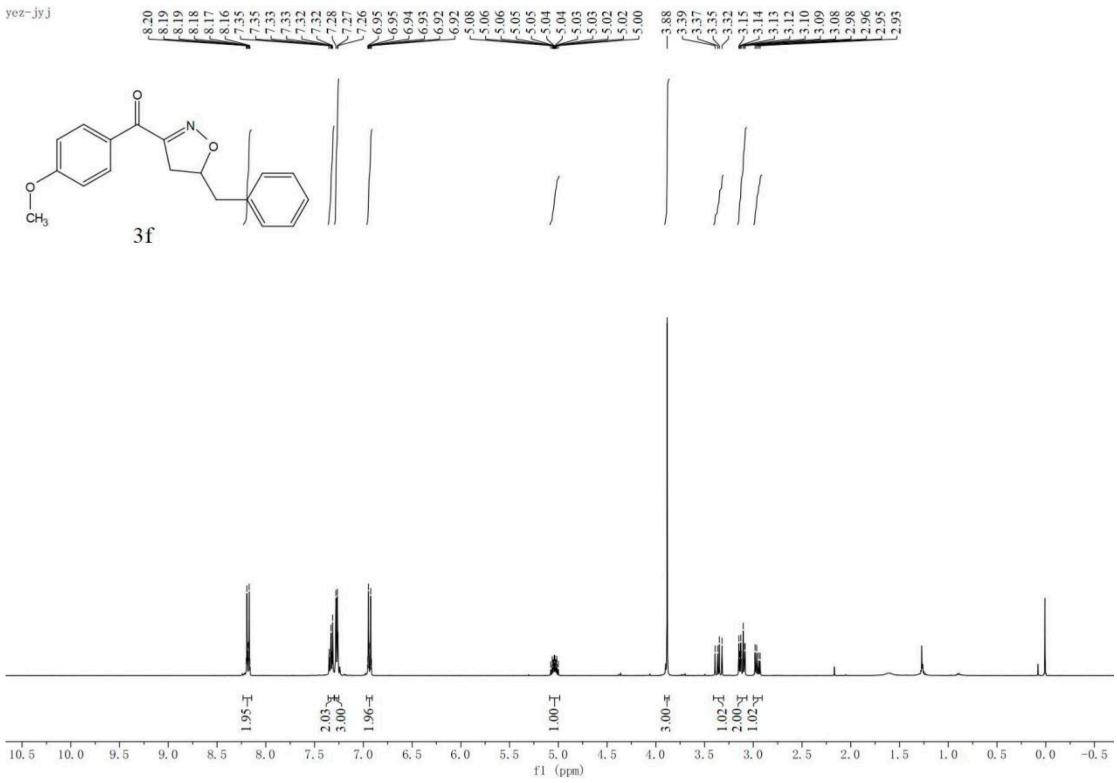
yez-jyj
8.20 8.19 8.19 8.18 8.17 8.16 7.35 7.35 7.33 7.33 7.32 7.32 7.28 7.27 7.26 6.95 6.95 6.94 6.93 6.93 6.92 5.08 5.06 5.06 5.05 5.05 5.04 5.04 5.03 5.03 5.02 5.02 5.00 3.88 3.39 3.37 3.35 3.32 3.15 3.14 3.13 3.12 3.10 3.09 3.08 2.98 2.96 2.95 2.93
3f
1.95 2.03 3.00 1.96 1.00 3.00 1.02 2.00 1.02
f1 (ppm)

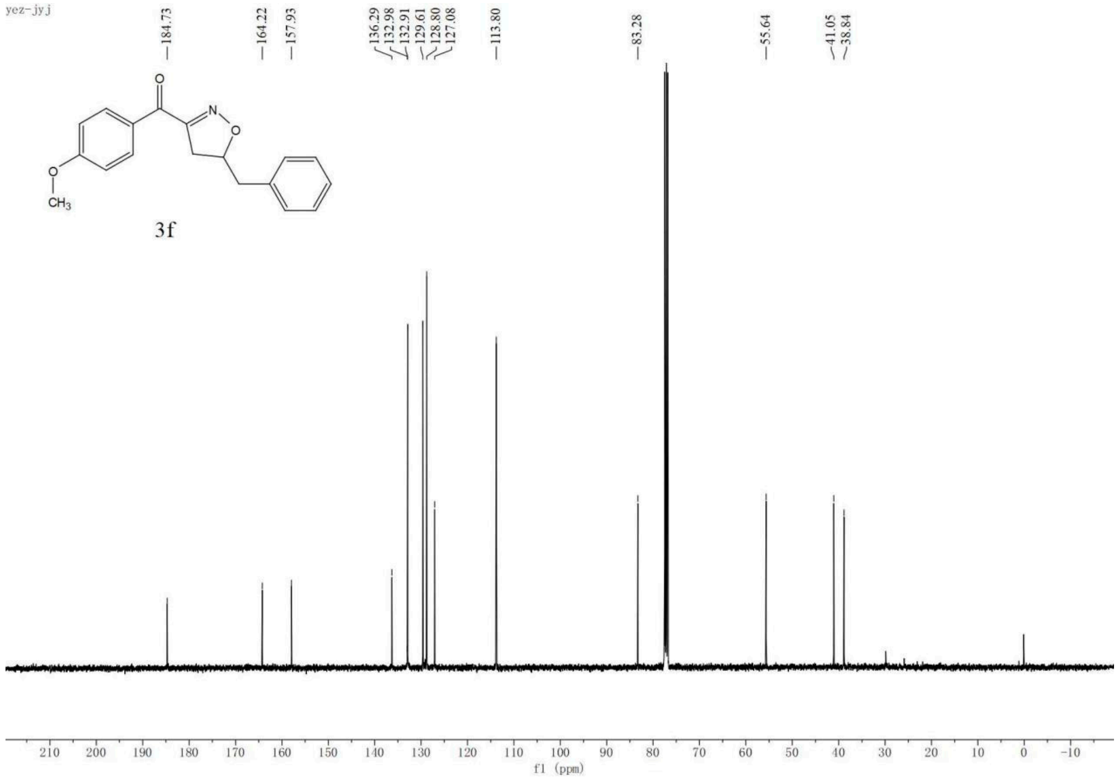
yez-jyj
184.73 164.22 157.93 136.29 132.98 132.91 129.61 128.80 127.08 113.80 83.28 55.64 41.05 38.84
3f
f1 (ppm)

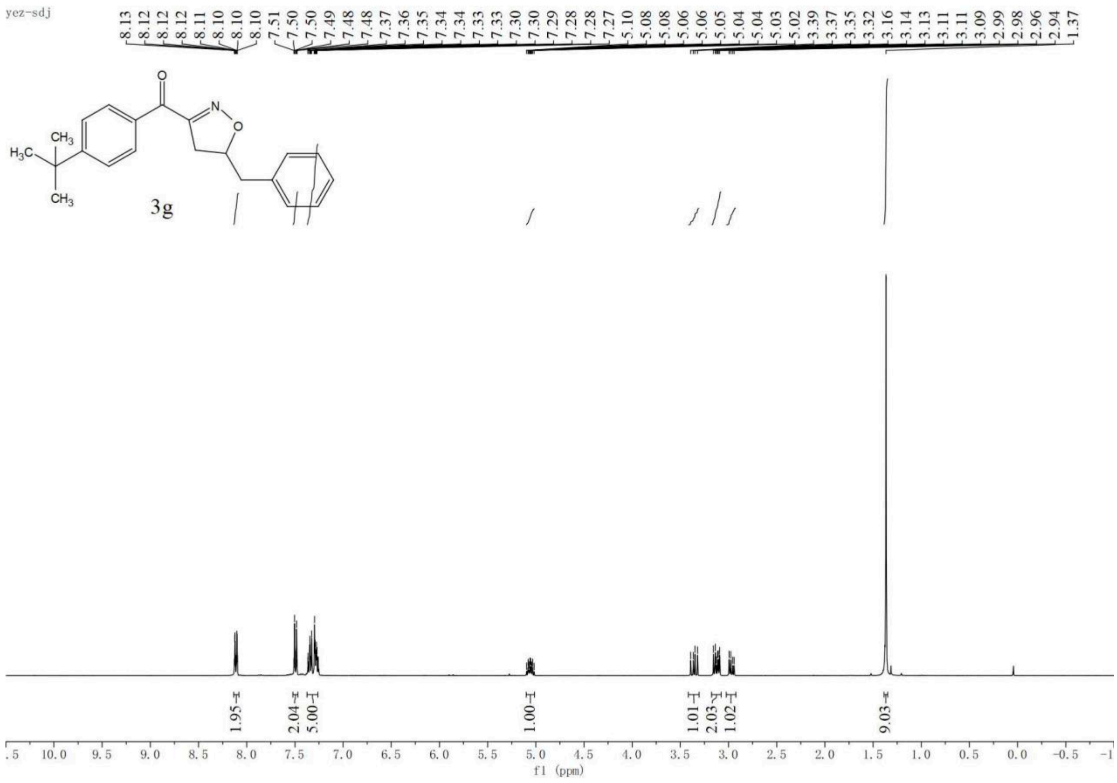
yez-sdj
8.13 8.12 8.12 8.12 8.11 8.10 8.10 8.10 7.51 7.50 7.50 7.49 7.48 7.48 7.37 7.36 7.35 7.34 7.34 7.33 7.33 7.30 7.30 7.29 7.28 7.28 7.27 5.10 5.08 5.06 5.06 5.05 5.04 5.04 5.03 5.02 3.39 3.37 3.35 3.32 3.16 3.14 3.13 3.11 3.09 2.99 2.98 2.96 2.94 1.37
3g
1.95 2.04 5.00 1.00 1.01 2.03 1.02 9.03
f1 (ppm)

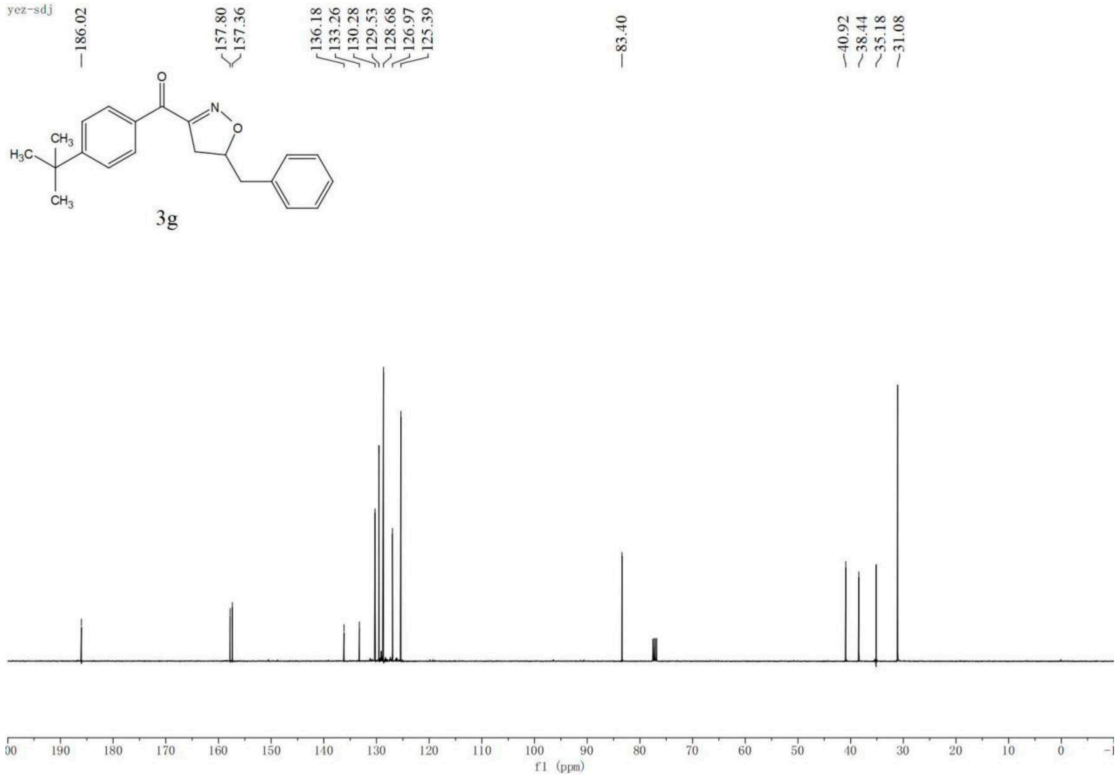
yez-sdj
186.02
157.80 157.36
136.18 133.26 130.28 129.53 128.68 126.97 125.39
83.40
40.92 38.44 35.18 31.08
3g
f1 (ppm)

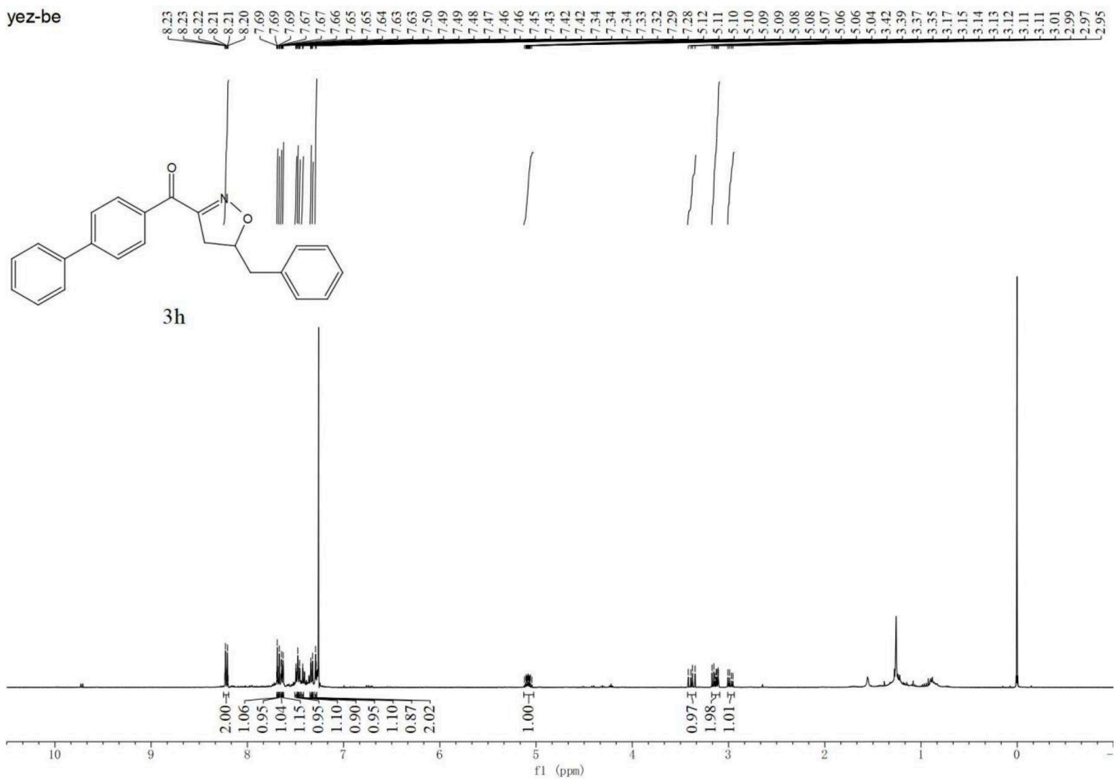
yez-be
8.23
8.23
8.22
8.21
8.21
8.20
7.69
7.69
7.69
7.67
7.66
7.65
7.65
7.64
7.63
7.63
7.50
7.49
7.49
7.48
7.47
7.46
7.46
7.45
7.43
7.42
7.42
7.34
7.34
7.34
7.33
7.32
7.29
7.28
5.12
5.11
5.10
5.10
5.09
5.09
5.08
5.08
5.07
5.06
5.06
5.04
3.42
3.39
3.37
3.35
3.17
3.15
3.14
3.13
3.12
3.11
3.11
3.01
2.99
2.97
2.95
O
N
O
3h
2.00
1.06
0.95
1.04
1.15
0.95
1.10
0.90
0.95
1.10
0.87
2.02
1.00
0.97
1.98
1.01
10
9
8
7
6
5
4
3
2
1
0
f1 (ppm)

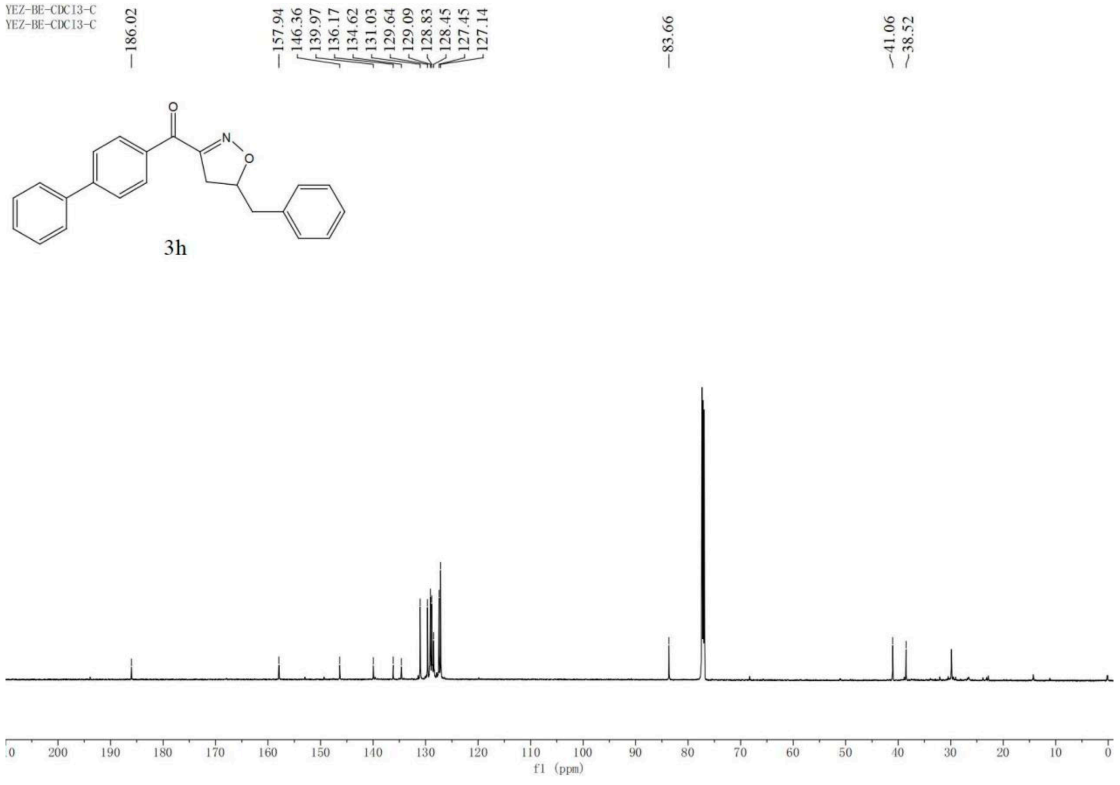
YEZ-BE-CDCl3-C
YEZ-BE-CDCl3-C
186.02
157.94
146.36
139.97
136.17
134.62
131.03
129.64
129.09
128.83
128.45
127.45
127.14
83.66
41.06
38.52
O
N
O
3h
200
190
180
170
160
150
140
130
120
110
100
90
80
70
60
50
40
30
20
10
0
f1 (ppm)

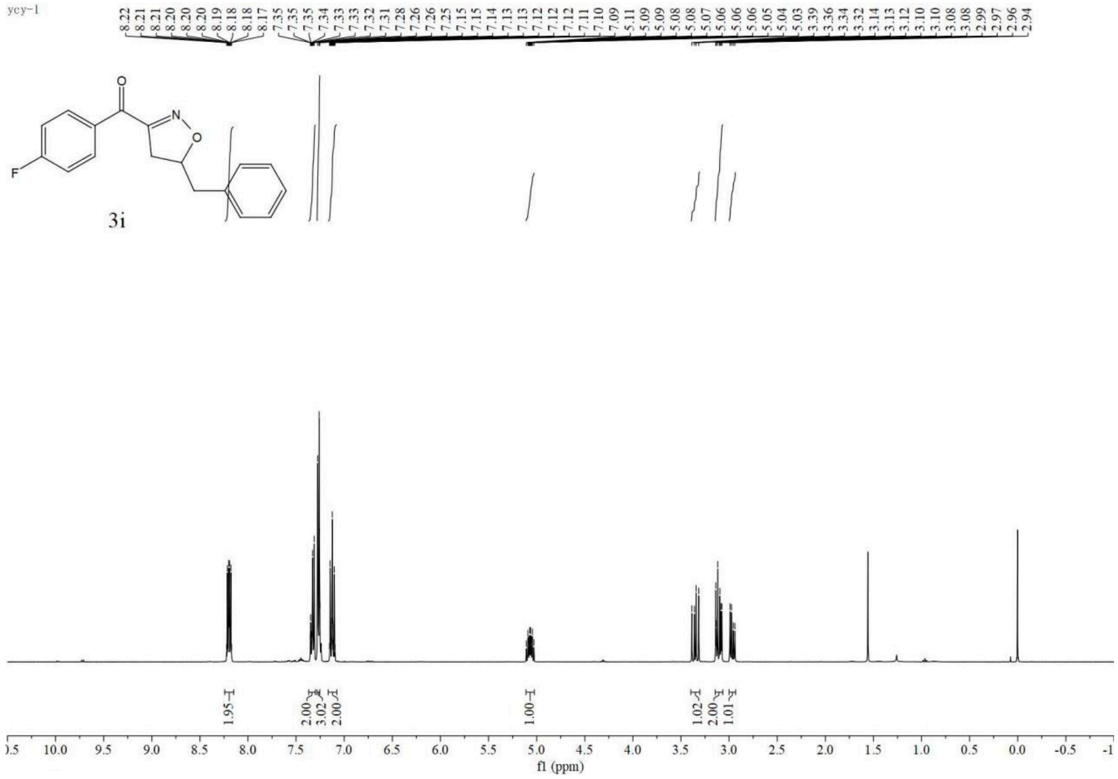
ycy-1
8.22 8.21 8.21 8.20 8.20 8.19 8.18 8.18 8.17 7.35 7.35 7.34 7.33 7.33 7.32 7.31 7.28 7.26 7.25 7.15 7.15 7.14 7.13 7.13 7.12 7.12 7.11 7.10 7.09 5.11 5.09 5.09 5.08 5.08 5.07 5.06 5.06 5.06 5.05 5.05 5.04 5.03 3.39 3.36 3.34 3.32 3.14 3.13 3.12 3.10 3.10 3.08 3.08 2.99 2.97 2.96 2.94
O
N
O
F
3i
1.95
2.00
3.02
2.00
1.00
1.02
2.00
1.01
fl (ppm)

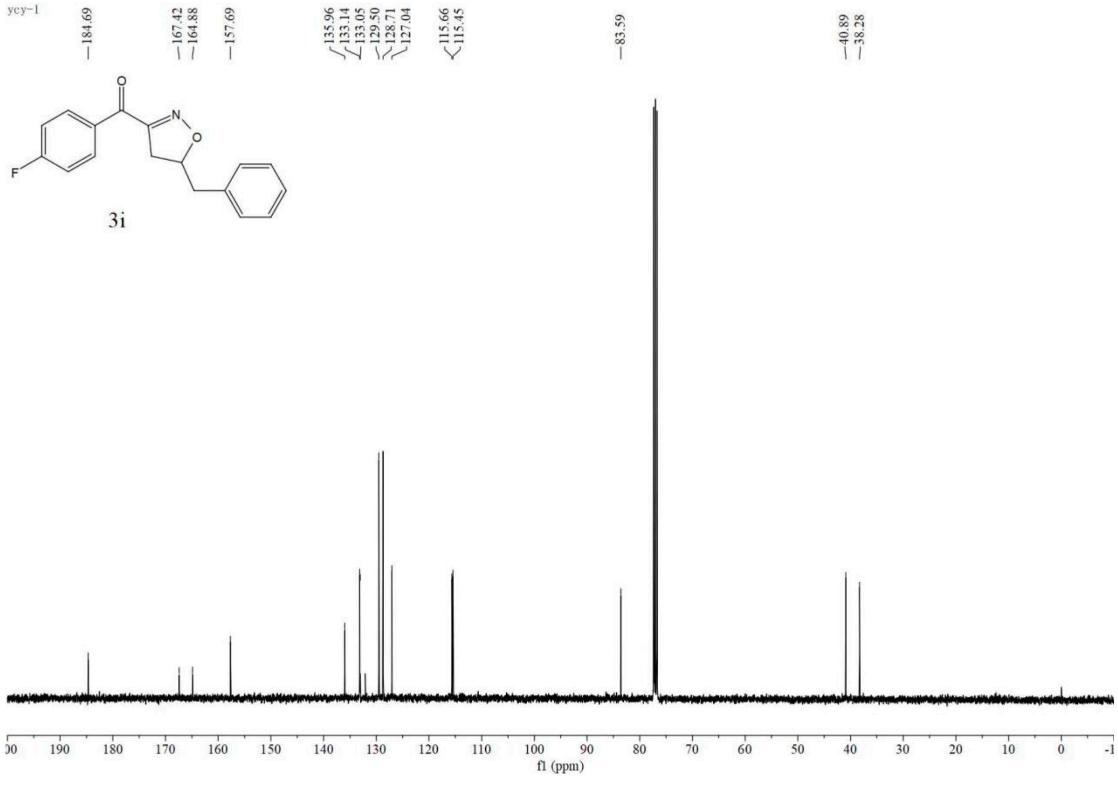
ycy-1
184.69
167.42
164.88
157.69
135.96
133.14
133.05
129.50
128.71
127.04
115.66
115.45
83.59
40.89
38.28
O
N
O
F
3i
fl (ppm)

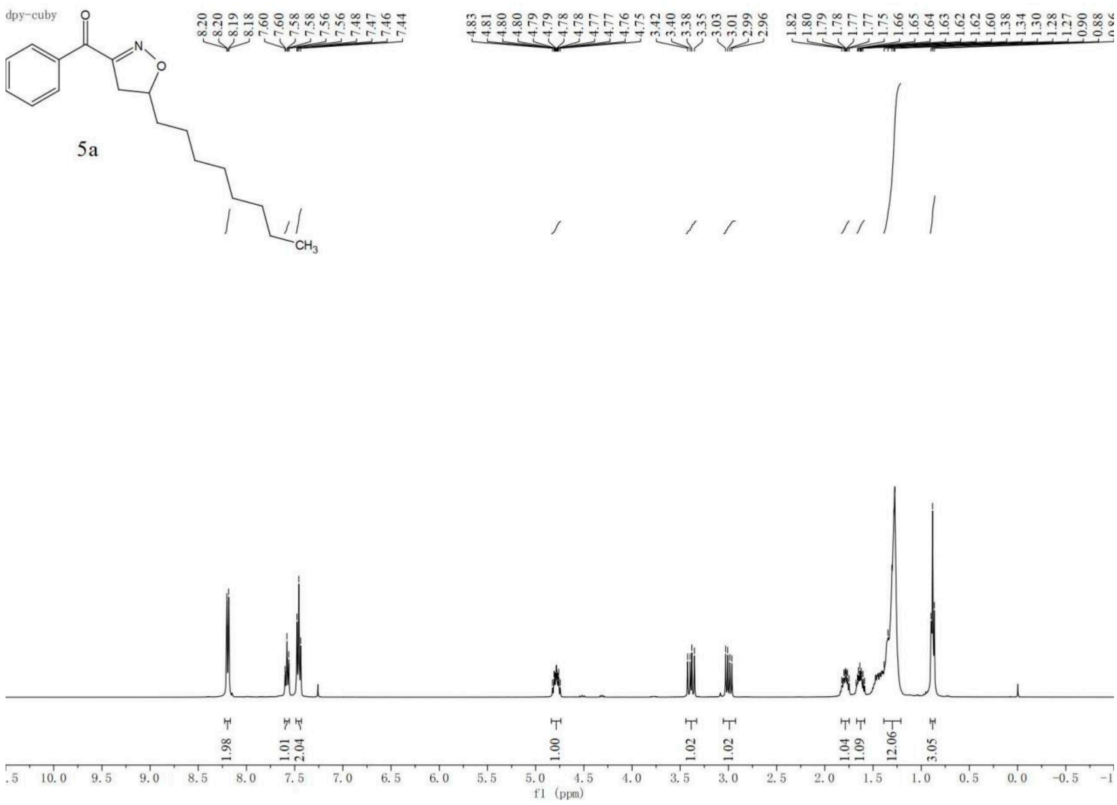
dpy-cuby
5a
f1 (ppm)

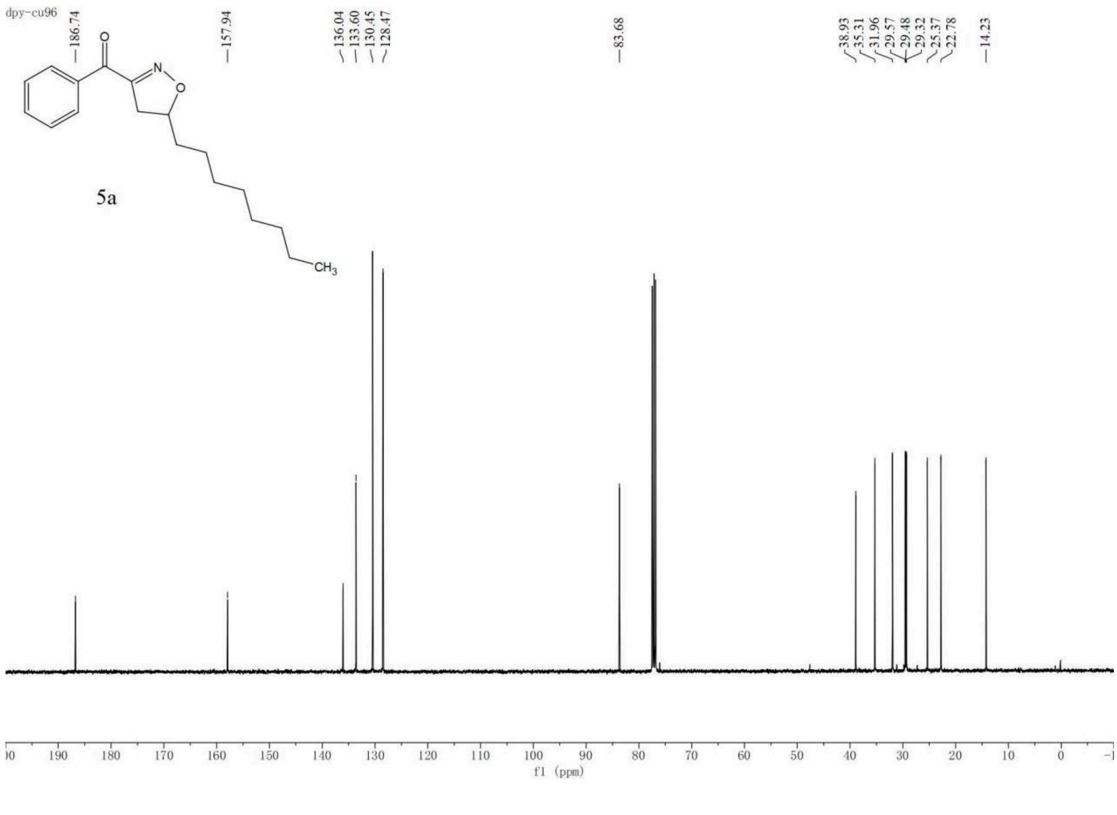
dpy-cu96
5a
f1 (ppm)

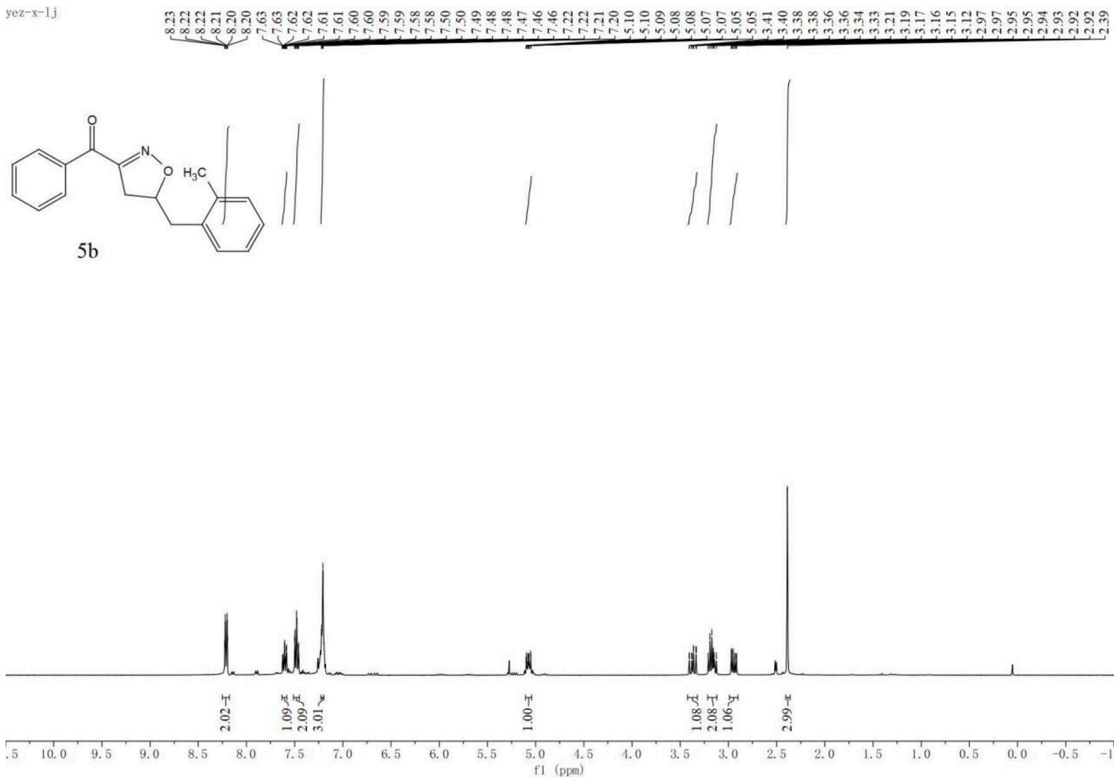
yez-x-1j
8.23 8.22 8.22 8.21 8.20 8.20 7.63 7.62 7.62 7.61 7.61 7.60 7.60 7.59 7.59 7.58 7.58 7.50 7.50 7.49 7.48 7.48 7.47 7.46 7.22 7.22 7.21 7.20 5.10 5.10 5.09 5.08 5.08 5.07 5.07 5.05 5.05 3.41 3.40 3.38 3.38 3.36 3.34 3.33 3.21 3.19 3.17 3.16 3.15 3.12 2.97 2.97 2.95 2.95 2.94 2.93 2.92 2.92 2.39
O
N
O
H_3C
5b
2.02
1.09
2.09
3.01
1.00
1.08
2.08
1.06
2.99
f1 (ppm)

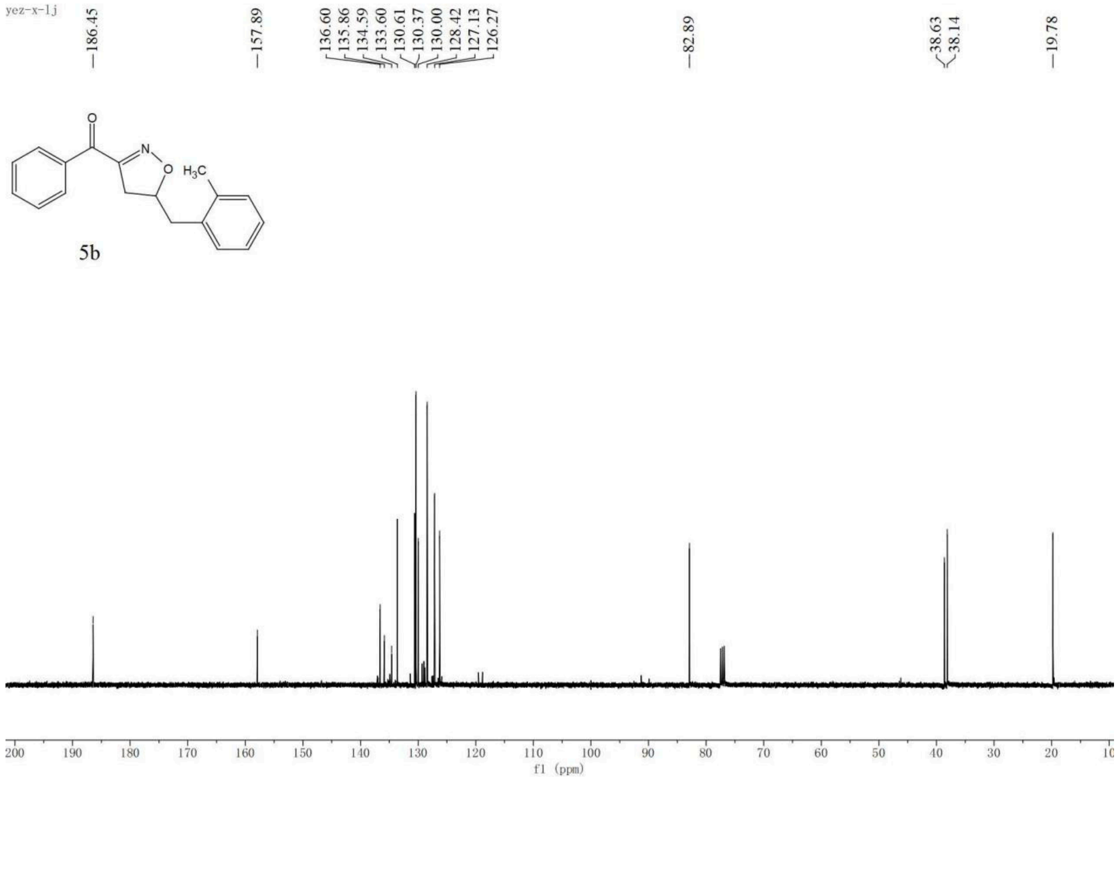
yez-x-1j
186.45
157.89
136.60 135.86 134.59 133.60 130.61 130.37 130.00 128.42 127.13 126.27
82.89
38.63 38.14
19.78
O
N
O
H_3C
5b
f1 (ppm)

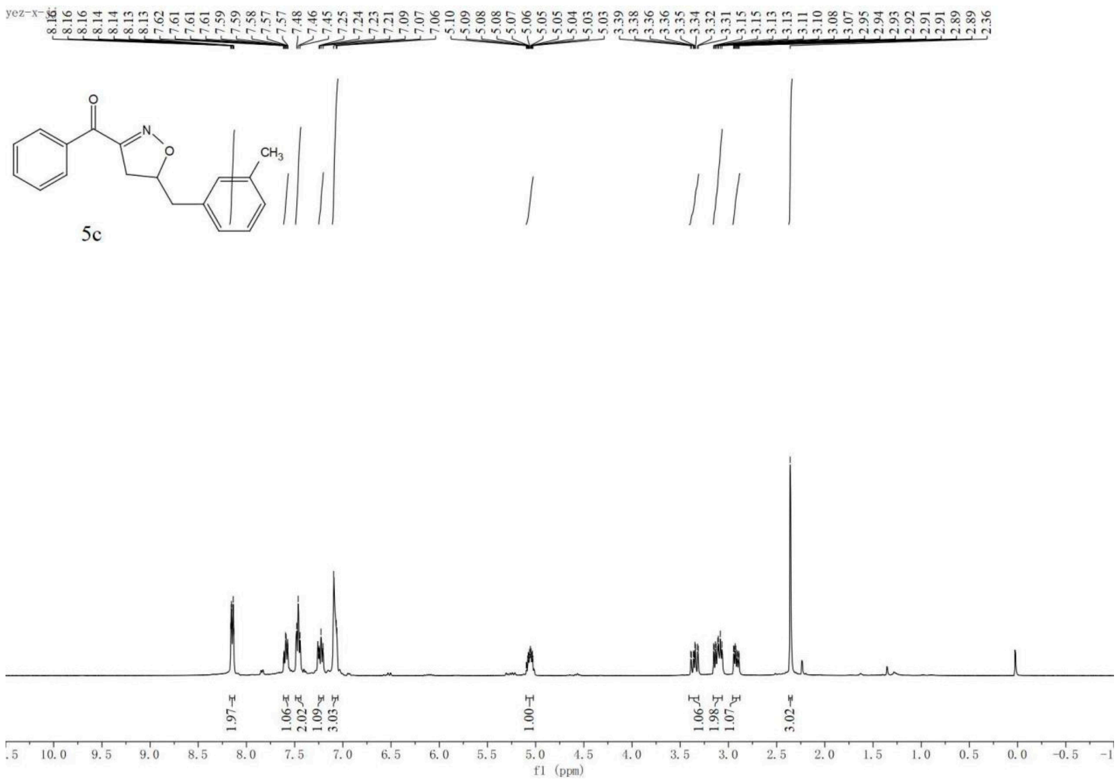
yez-x-jj
8.16 8.16 8.16 8.14 8.14 8.13 8.13 7.62 7.61 7.61 7.61 7.59 7.59 7.58 7.57 7.57 7.48 7.46 7.45 7.25 7.24 7.23 7.21 7.09 7.07 7.06 5.10 5.09 5.08 5.07 5.06 5.05 5.05 5.04 5.03 5.03 3.39 3.38 3.36 3.35 3.34 3.32 3.31 3.15 3.15 3.13 3.13 3.11 3.10 3.08 3.07 2.95 2.94 2.93 2.92 2.91 2.91 2.89 2.89 2.36
5c
1.97 1.06 2.02 1.09 3.03 1.00 1.06 1.98 1.07 3.02
f1 (ppm)

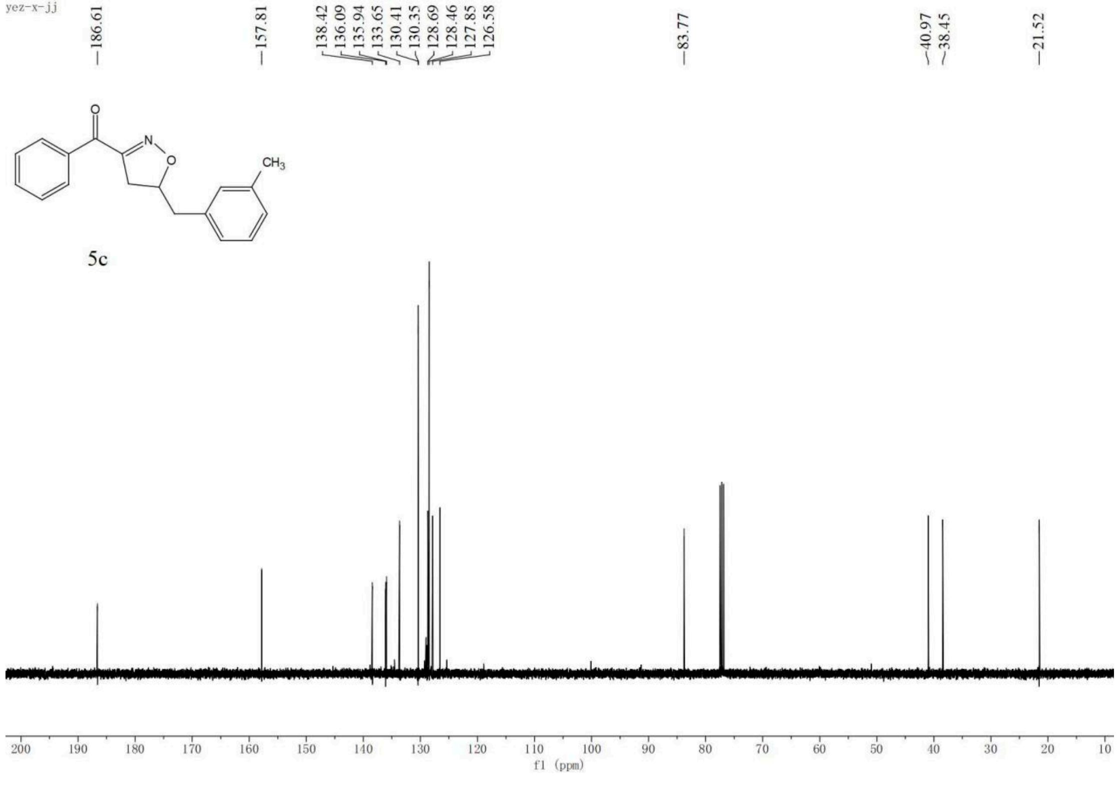
yez-x-jj
186.61 157.81 138.42 136.09 135.94 133.65 130.41 130.35 128.69 128.46 127.85 126.58 83.77 40.97 38.45 21.52
5c
f1 (ppm)

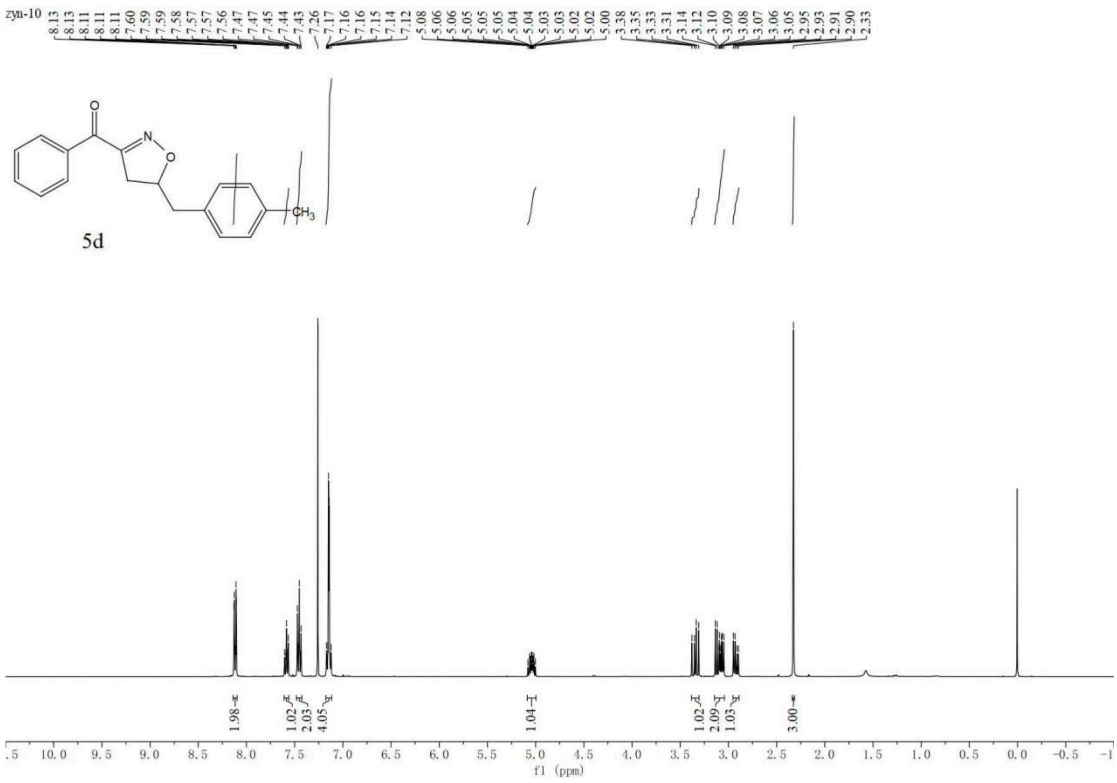
zyn-10
8.13 8.13 8.11 8.11 7.60 7.59 7.59 7.58 7.57 7.57 7.56 7.47 7.47 7.45 7.44 7.43 7.26 7.17 7.16 7.15 7.14 7.12 5.08 5.06 5.06 5.05 5.05 5.05 5.04 5.04 5.03 5.03 5.02 5.02 5.00 3.38 3.35 3.33 3.31 3.14 3.12 3.10 3.09 3.08 3.07 3.06 3.05 2.95 2.93 2.91 2.90 2.33
O
N
O
CH3
5d
1.98
1.02
2.03
4.05
1.04
1.02
2.09
1.03
3.00
.5 10.0 9.5 9.0 8.5 8.0 7.5 7.0 6.5 6.0 5.5 5.0 4.5 4.0 3.5 3.0 2.5 2.0 1.5 1.0 0.5 0.0 -0.5 -1
f1 (ppm)

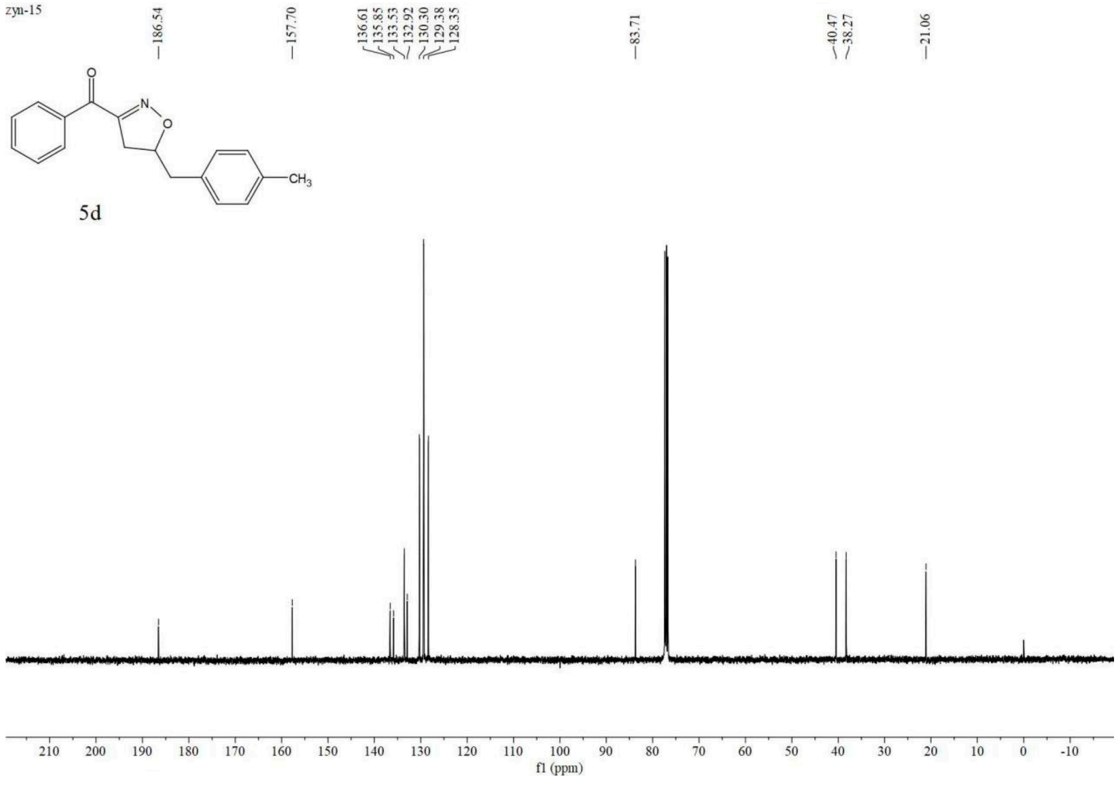
zyn-15
186.54
157.70
136.61 135.85 133.53 132.92 130.30 129.38 128.35
83.71
40.47 38.27
21.06
O
N
O
CH3
5d
210 200 190 180 170 160 150 140 130 120 110 100 90 80 70 60 50 40 30 20 10 0 -10
f1 (ppm)

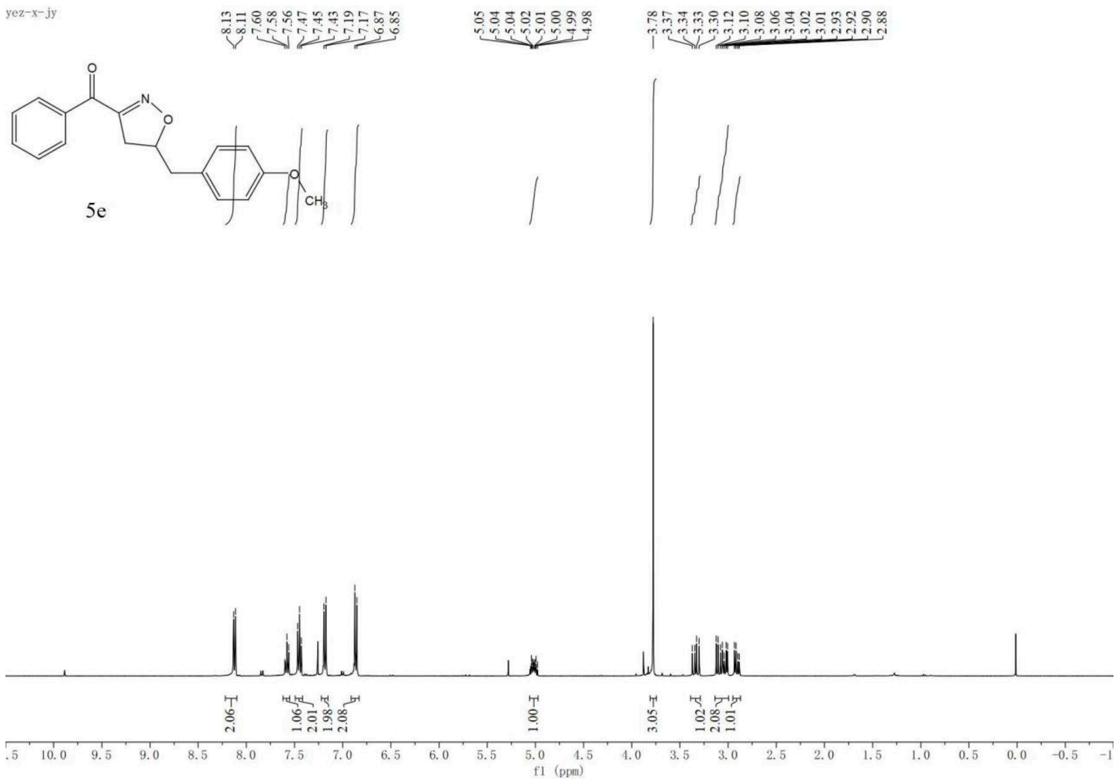
yez-x-jy
8.13
8.11
7.60
7.58
7.56
7.47
7.45
7.43
7.19
7.17
6.87
6.85
5.05
5.04
5.04
5.02
5.01
5.00
4.99
4.98
3.78
3.37
3.34
3.33
3.30
3.12
3.10
3.08
3.06
3.04
3.02
3.01
2.93
2.92
2.90
2.88
O
N
O
O
CH3
5e
2.06
1.06
2.01
1.98
2.08
1.00
3.05
1.02
2.08
1.01
f1 (ppm)

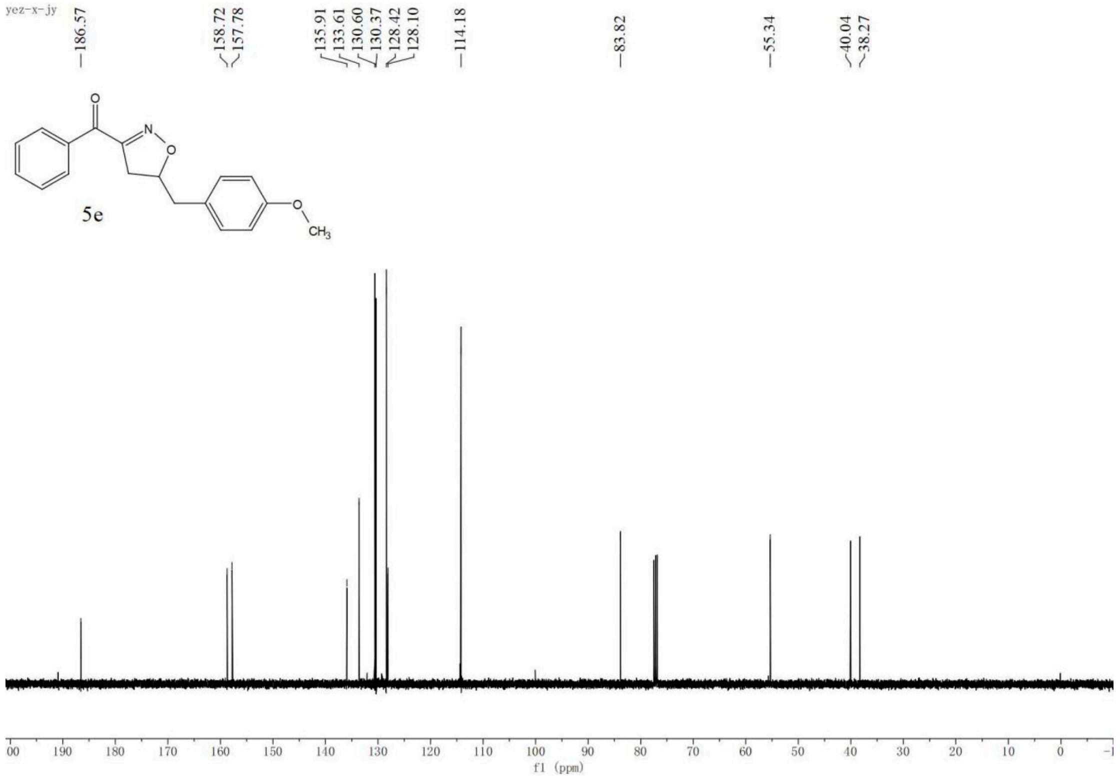
yez-x-jy
186.57
158.72
157.78
135.91
133.61
130.60
130.37
128.42
128.10
114.18
83.82
55.34
40.04
38.27
O
N
O
O
CH3
5e
f1 (ppm)

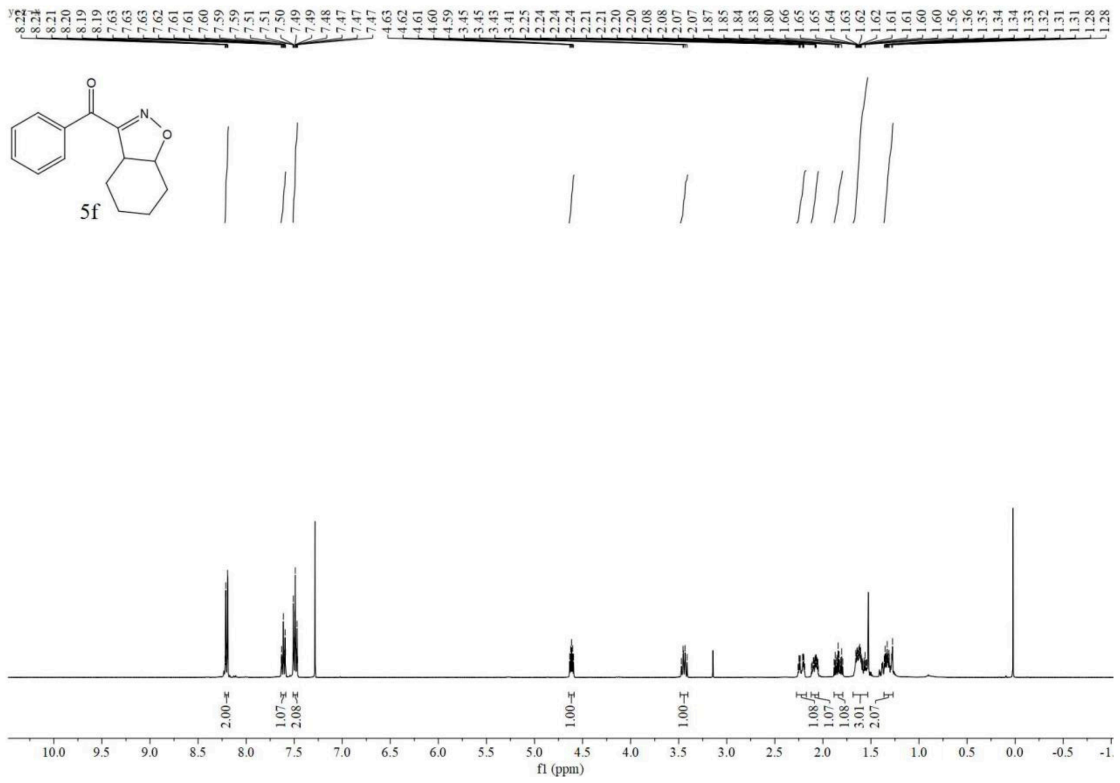
5f
2.00
1.07
2.08
1.00
1.00
1.08
1.07
1.08
3.01
2.07
f1 (ppm)

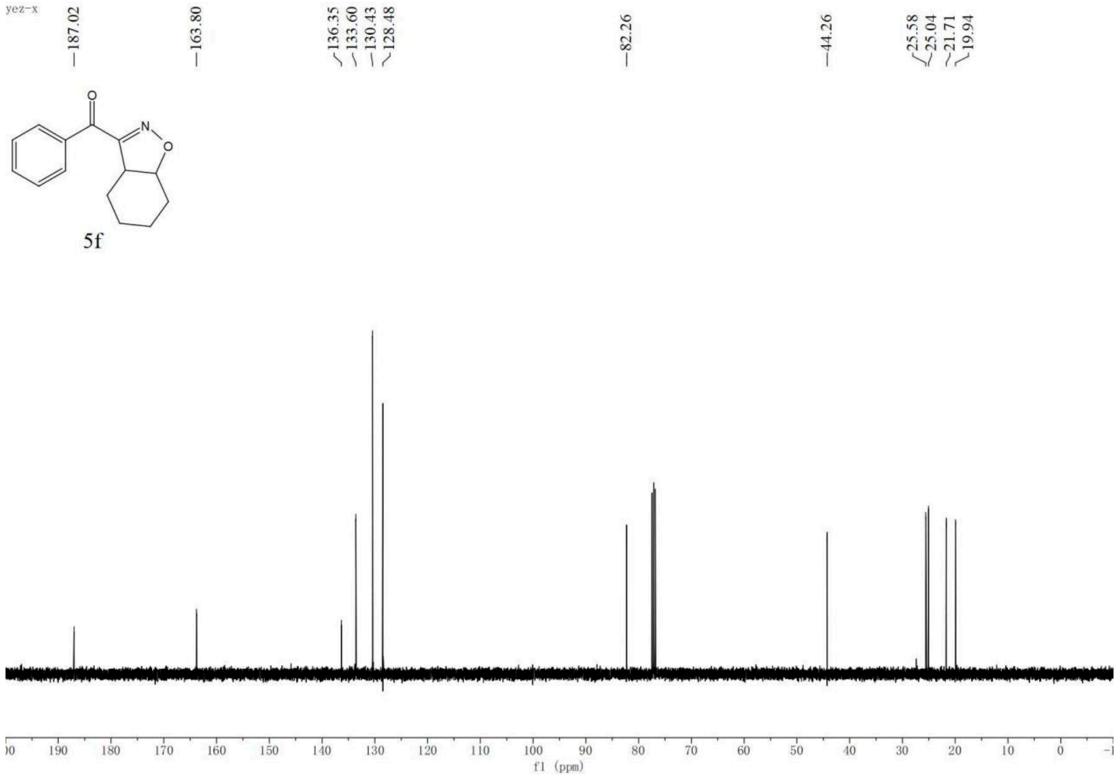
yez-x
187.02
163.80
136.35
133.60
130.43
128.48
82.26
44.26
25.58
25.04
21.71
19.94
5f
f1 (ppm)

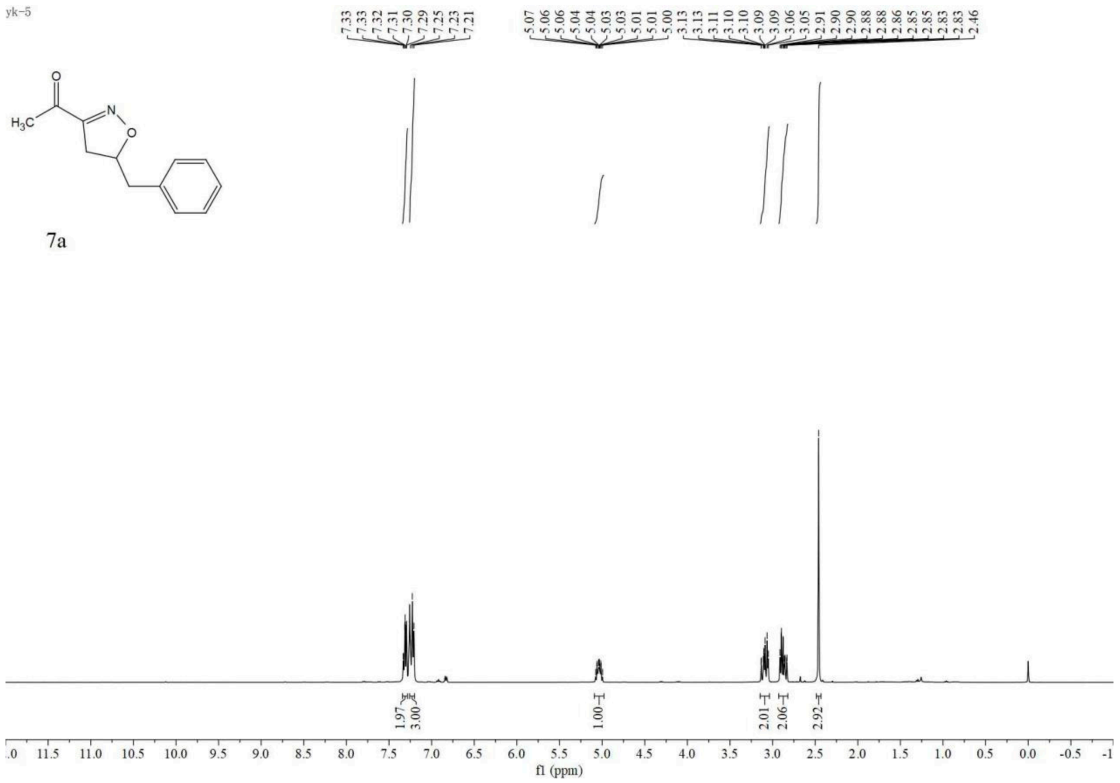
yk-5
7.33 7.33 7.32 7.31 7.30 7.29 7.25 7.23 7.21
5.07 5.06 5.06 5.04 5.04 5.03 5.03 5.01 5.01 5.00
3.13 3.13 3.11 3.10 3.10 3.09 3.09 3.06 3.05 2.91 2.90 2.90 2.88 2.88 2.86 2.85 2.85 2.83 2.83 2.46
H3C
N
O
7a
1.97
3.00
1.00
2.01
2.06
2.92
11.5 11.0 10.5 10.0 9.5 9.0 8.5 8.0 7.5 7.0 6.5 6.0 5.5 5.0 4.5 4.0 3.5 3.0 2.5 2.0 1.5 1.0 0.5 0.0 -0.5
f1 (ppm)

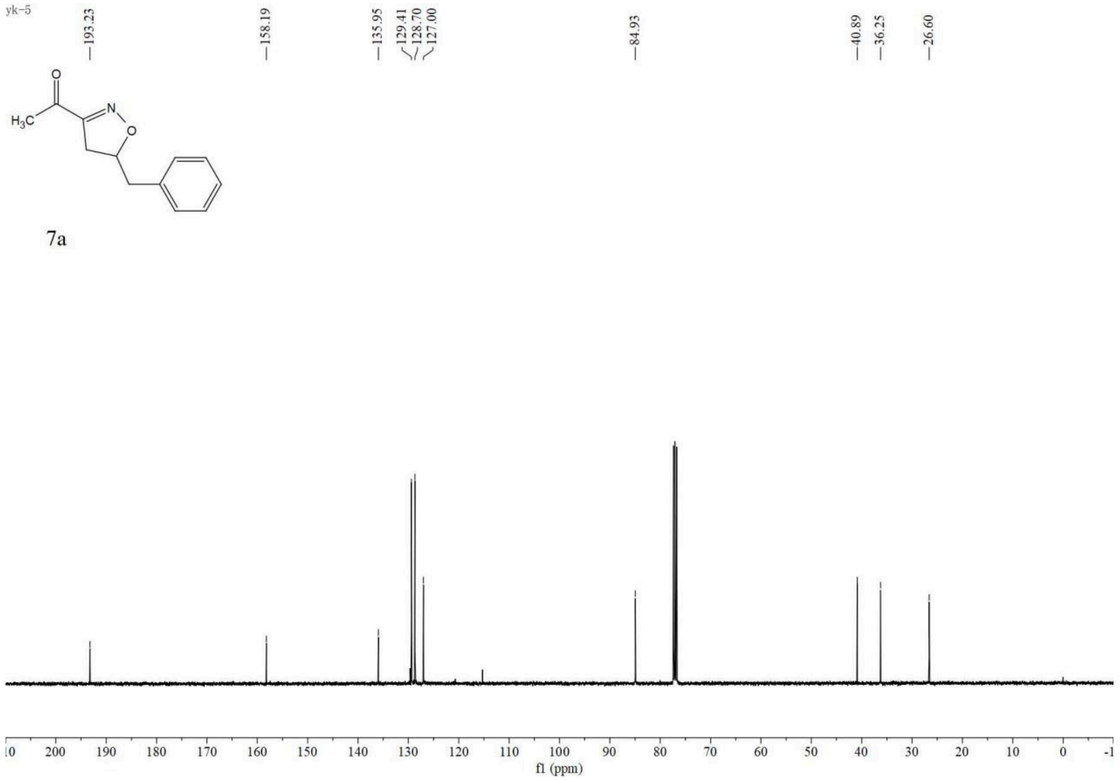
yk-5
193.23
158.19
135.95
129.41
128.70
127.00
84.93
40.89
36.25
26.60
H3C
N
O
7a
200 190 180 170 160 150 140 130 120 110 100 90 80 70 60 50 40 30 20 10 0
f1 (ppm)

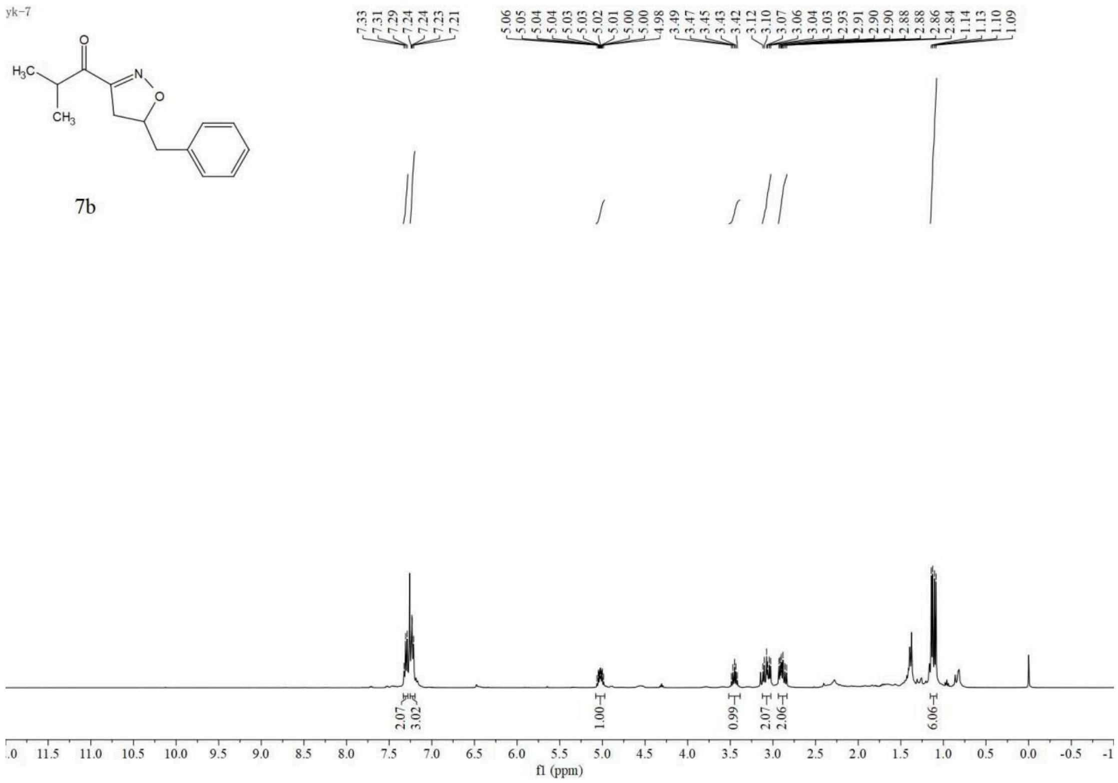
yk-7
7.33
7.31
7.29
7.24
7.24
7.23
7.21
5.06
5.05
5.04
5.04
5.03
5.03
5.02
5.01
5.00
5.00
4.98
3.49
3.47
3.45
3.43
3.42
3.12
3.10
3.07
3.06
3.04
3.03
2.93
2.91
2.90
2.90
2.88
2.88
2.86
2.84
1.14
1.13
1.10
1.09
7b
2.07
3.02
1.00
0.99
2.07
2.06
6.06
f1 (ppm)

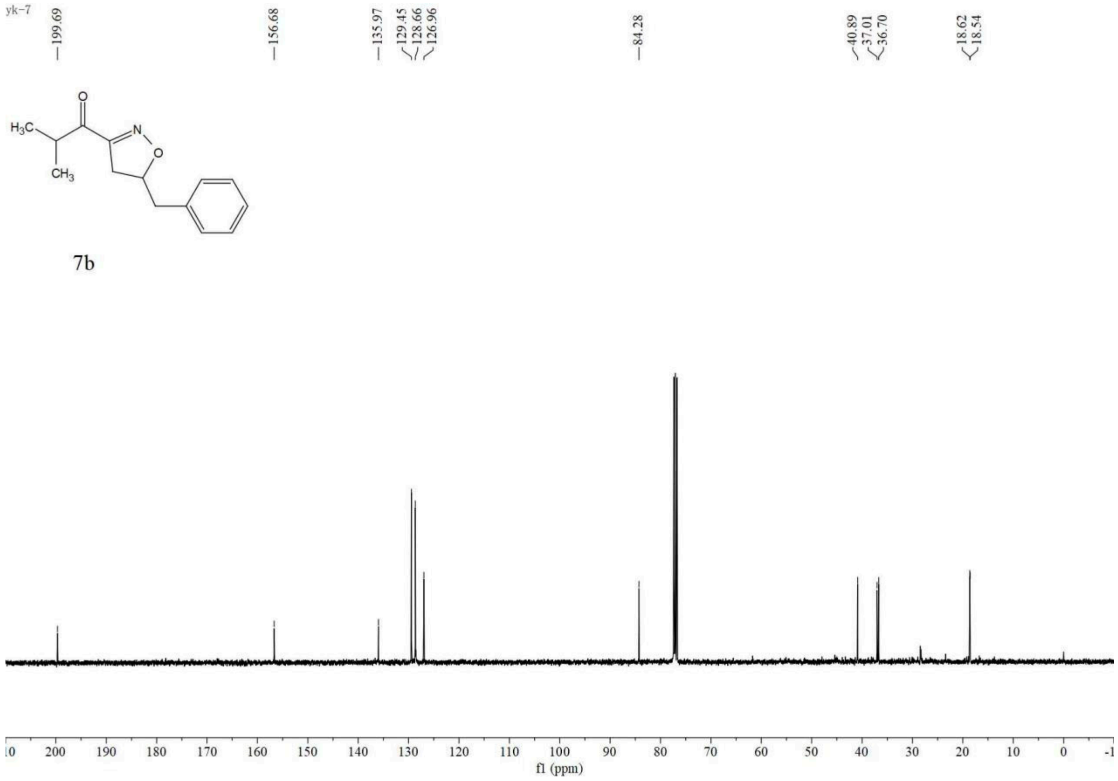
yk-7
199.69
156.68
135.97
129.45
128.66
126.96
84.28
40.89
37.01
36.70
18.62
18.54
7b
f1 (ppm)

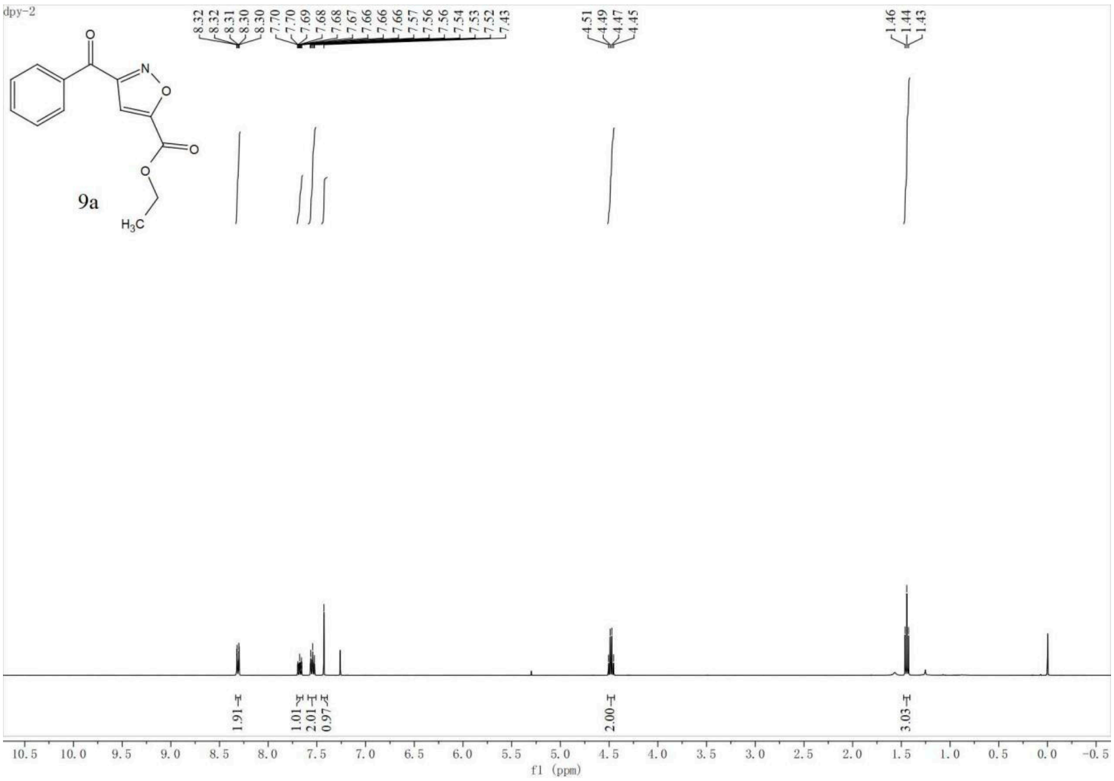
dpy-2
9a
8.32
8.32
8.31
8.30
8.30
7.70
7.70
7.69
7.68
7.68
7.67
7.66
7.66
7.66
7.57
7.56
7.56
7.54
7.53
7.52
7.43
4.51
4.49
4.47
4.45
1.46
1.44
1.43
1.91
1.01
2.01
0.97
2.00
3.03
f1 (ppm)

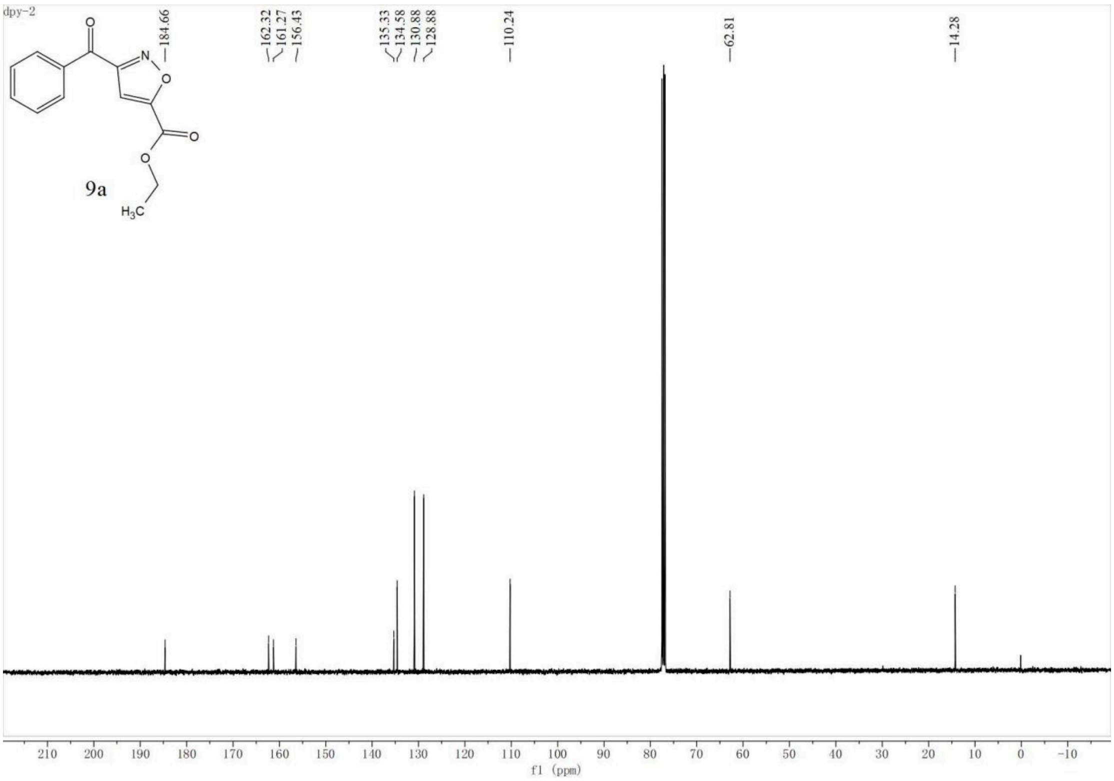
dpy-2
9a
184.66
162.32
161.27
156.43
135.33
134.58
130.88
128.88
110.24
62.81
14.28
f1 (ppm)

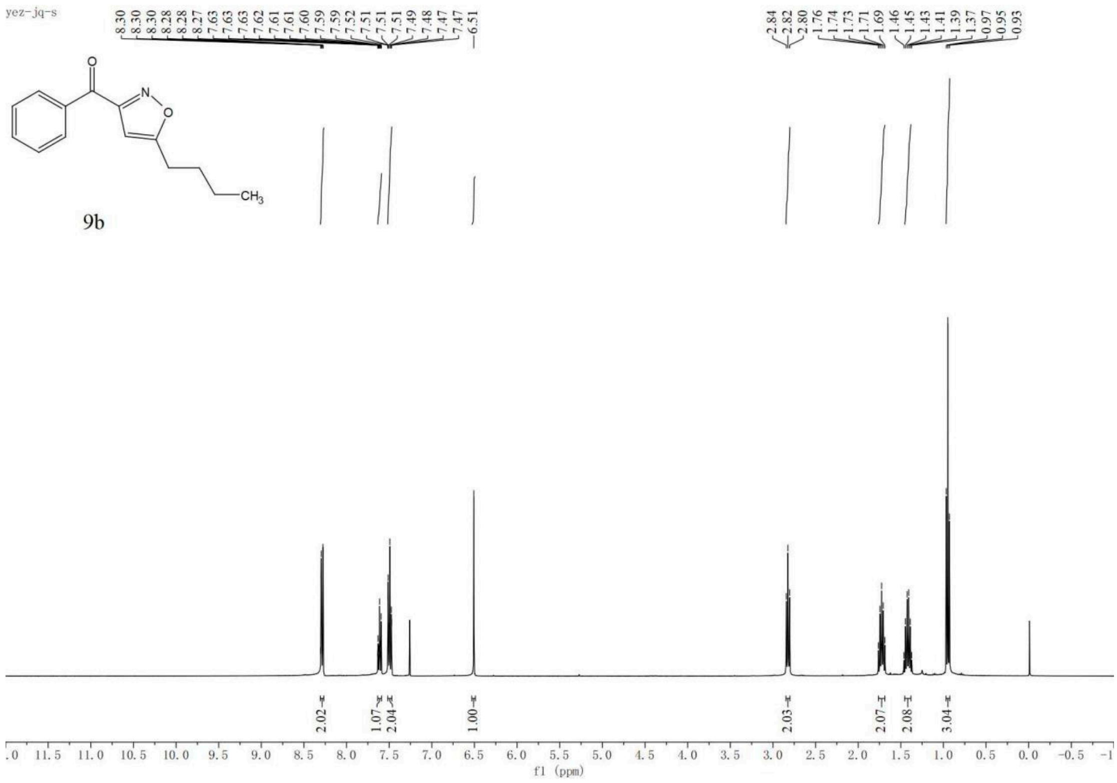
yez-jq-s
8.30
8.30
8.30
8.28
8.28
8.27
7.63
7.63
7.63
7.62
7.61
7.61
7.60
7.59
7.59
7.52
7.51
7.51
7.51
7.49
7.48
7.47
7.47
6.51
2.84
2.82
2.80
1.76
1.74
1.73
1.71
1.69
1.46
1.45
1.43
1.41
1.39
1.37
0.97
0.95
0.93
9b
2.02
1.07
2.04
1.00
2.03
2.07
2.08
3.04
f1 (ppm)

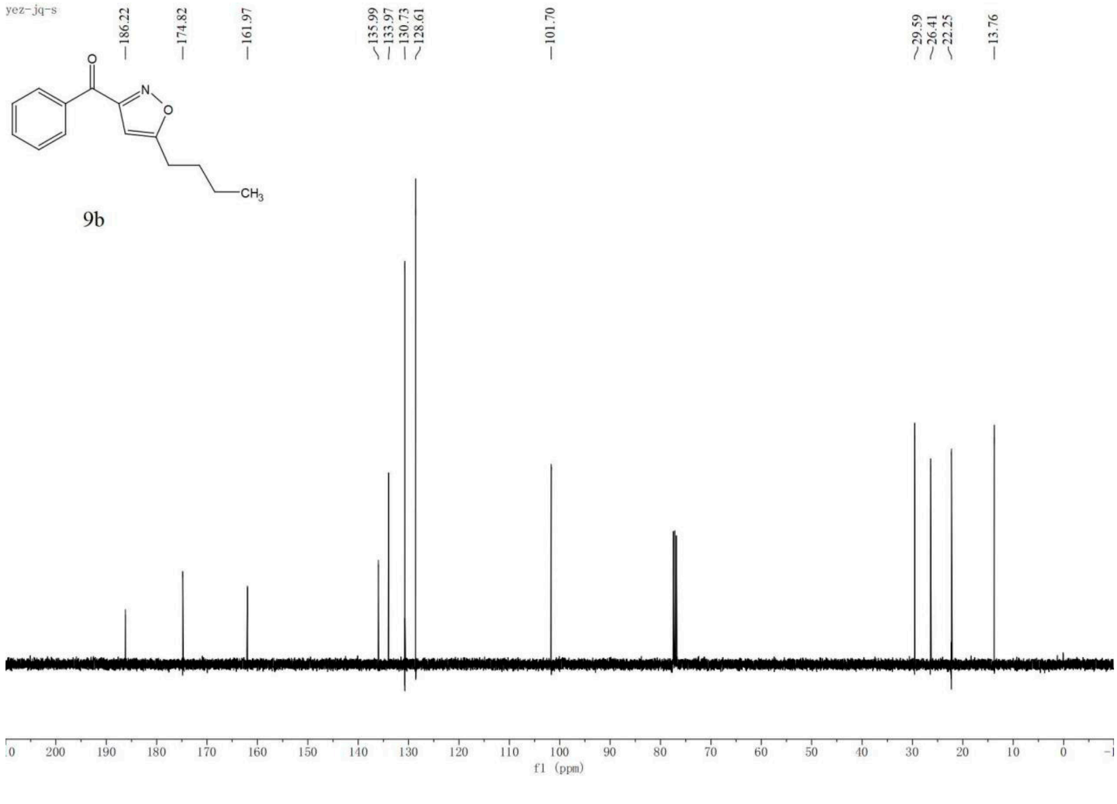
yez-jq-s
186.22
174.82
161.97
135.99
133.97
130.73
128.61
101.70
29.59
26.41
22.25
13.76
9b
f1 (ppm)
